# Developing students’ reading competence in an EFL context through annotated reading: A blended instructional design

**DOI:** 10.1371/journal.pone.0357895

**Published:** 2026-10-14

**Authors:** Yinxia Guan, Haomin Zhang

**Affiliations:** 1 School of Foreign Languages, Guangdong Polytechnic Normal University, Guangzhou, Guangdong, China; 2 Faculty of Humanities and Social Sciences, City University of Macau, Macau SAR, China; 3 The Psycholinguistics Lab, School of Foreign Languages, East China Normal University, Shanghai, China; Xi’an Jiaotong-Liverpool University, CHINA

## Abstract

Annotated reading is generally believed an effective way to improve learners’ reading competence. However, most existing studies focus on online annotated reading while neglecting to scaffold students throughout the entire learning process. To address the gap, this study proposed a four-stage annotated reading framework and employed a quasi-experimental design to evaluate its effectiveness in developing students’ reading competence. Concurrently, students’ perceptions and actualization of this pedagogy were also investigated. Results demonstrated that this framework had potential in cultivating students’ reading competence, it was perceived as offering substantially more benefits than difficulties, and students who yielded greater gains exhibited higher engagement levels. Furthermore, modes of learning were not the decisive factor in learning outcomes, and students’ engagement proved equally critical. The findings can facilitate the field’s re-examination of technology use and shifting more focus to students’ individual differences and the holistic teaching-learning process. Targeted pedagogical suggestions were presented based on the findings.

## 1. Introduction

Reading competence, the ability to read and understand what has been read, is considered a key competence that remains important throughout a person’s life [1], and ranked among the most pivotal language skills in language education [2]. In English learning, reading can not only expand learners’ vocabulary [3,4] but also improve their language output abilities [5], and the transfer of reading skills can be applied to other language skills such as listening, speaking and writing [6]. Therefore, developing students’ English reading competence is one of the prominent responsibilities for English teachers.

Annotated reading is believed to be effective in improving learners’ reading competence [7] and academic performance [8] due to their active engagement with texts thus resulting in deeper understanding and longer memory retention. With the development of Internet and modern technology, the medium of reading has shifted from paper-based reading towards online reading [9], and a variety of digital annotation tools such as Diigo, Hypothesis and Perusall mushroom. It makes social reading, an online approach to reading that integrates social, interpersonal, and interactive elements in a collaborative and constructive process [10,11], possible. These tools provide platforms for readers not only to read and annotate but also to share, discuss or even dispute, and hence foster social interaction. To date, social annotated reading has been reported to provide contextual, social, and linguistic affordances for EFL learners [12], reinforce learner engagement [13,14], enhance reading motivation [15,16] and bring positive affect [16].

However, despite its prosperity, there also exist findings reporting deficiencies of solely relying on online annotated reading. Paper-based annotated reading demonstrated merits over its online counterpart for it gives readers the opportunity to slow down and add their own thoughts and comments, raise questions for further investigation, and challenge ideas within the text [17] while relieving the burden of hardware requirement [18]. Furthermore, both Aydogdu and Winder [19], and Chen and Chen [20] reported that face-to-face interaction was superior because it fostered better group dynamics, participation, motivation, and deeper learning compared to online modes.

To make full use of the pedagogical benefits of paper-based annotated reading, offline group interaction and social reading, we designed a blended four-stage annotated reading instructional framework. A quasi-experiment with mixed research methods was conducted in an EFL context to verify its impact on students’ reading competence as well as explore students’ perceptions of and actualization of their annotated reading practices. The findings would have implications on annotated reading instruction and it is also our hope that this instructional design will help inform future studies.

## 2. Literature review

### 2.1. Annotated reading and pedagogical frameworks

Annotated reading, also known as close reading, is a deliberate interaction with a text with the aim of helping the reader to understand it more fully [17]. Sustained attention can be conceptualized as the core cognitive underpinning for annotated reading. Öksüz et al. [21] demonstrate that sustained attention serves as a “cognitive bridge” during reading—it not only directly predicts comprehension levels but also acts as a full mediator, transforming the efficacy of verbal working memory into accessible reading comprehension ability. Thus, the practice of annotated reading—which requires readers to continuously select, mark, and reflect on text segments—can be seen as a natural behavioral expression of sustained attention, training the very process of consistently and strategically allocating attention to key information.

Over the past two decades, although social annotated reading has been widely integrated into educational settings, its instructional design has often been limited to providing learners with basic annotation tools, such as highlighting, underlining, or free-form commenting, without further pedagogical refinement [22–24]. Thus, students may either lack the motivation to wrestle with intricate perspectives or lack necessary capabilities in navigating complex ideas in social reading [25]. Fortunately, this gap has aroused researchers’ attention, more sophisticated teaching design has been attempted to scaffold students’ learning. In the EFL educational domain, three prominent research directions have emerged. One is guiding students to focus more on (meta)cognitive skills. For example, following the work of Zhu, Shui, and Chen [11], who introduced participatory roles (facilitator, synthesizer, and summarizer) for undergraduates, Xu and Dai [26] adapted the same framework for postgraduate students. Both studies confirmed the framework’s efficacy in fostering active participation and knowledge construction, with Xu and Dai further noting its amplified impact on learners possessing lower prior knowledge. Additionally, Yeh, Hung, and Chiang [27] and Tseng and Yeh [28] both employed reciprocal teaching (RT) strategies—predicting, clarifying, questioning, and summarizing—to support students’ reading comprehension, with both studies reporting promising results.The second direction is to optimize the learning process. For example, an “initialization–exploration–consolidation–celebration” procedure [29] was adopted to make the learning process more coherent and logical. Similarly, Kohnke and Har [14] adopted a four-stage procedure “orientation–scaffolding students’ reading–students’ collaboration–responsiveness and evaluation” to promote interaction and engagement. The third one focuses on technological innovation by introducing new features into social annotation (SA) platforms, such as infusing gamification mechanisms [30] or formative assessment and feedback mechanisms [31] into collaborative reading system to motivate students’ annotation behaviors.

These attempts, while broadening the scope of annotated reading pedagogy, were all implemented on social reading platforms. Consequently, the focus remains predominantly on fostering engagement and interaction, with only a few specific reading skills being practiced. Reading competence is often defined as a multifaceted skill containing the following sub-skills: (1) cognitive abilities: vocabulary, grammatical understanding, the capacity to extract and integrate meaning from texts, and recognizing structures [32–34]; (2) meta-cognitive strategies: the ability to monitor comprehension, apply reading strategies, e.g., summarizing, questioning, and adjusting reading approaches as needed [35,36]; (3) critical evaluation: critically evaluating content and detecting bias [33,34]; (4) motivation and engagement: interest in reading, persistence, and a positive attitude toward reading [37]. Overall, existing interventions have addressed only a narrow range of sub-skills. Cultivating a broader spectrum requires more nuanced and purposefully designed annotation types.

Drawing on previous works, we adopted a blended design featuring paper-based annotation, offline discussion, SA, and class presentation/feedback while introducing a wider array of annotation types to train specific sub-skills, which aimed to holistically foster students’ reading competence.

### 2.2. The impact of annotated reading on students’ reading competence

Paper-based annotated reading, the early stage of annotated reading, has long been recognized as a useful way to improve learners’ reading competence and learning outcomes [8,17]. With the advancement of modern technology, the emergence of various SA tools and their application in education has made social annotated reading popular [38]. A number of studies endorsed its effects in improving learners’ reading comprehension [24,27,28,39]. However, there still existed literature reporting quite complicated or even contradictory results. Johnson et al. [22] discussed effects of Social Annotation Model-Learning System on students’ reading comprehension, critical thinking, and meta-cognitive skills with a sample of 254 American freshmen. The result showed that in individual learning there was no significant improvement in reading comprehension skills between the pre-test and post-test. Another research [40] indicated Chinese freshmen reading on Waiyanzaixian (a reading app with annotating functions) with their mobile-phones only showed marginal enhancements in reading comprehension accuracy, while paper-based reading group outperformed their counterparts both in reading comprehension accuracy and reading speed. Besides, paper readers were reported to perform better than digital readers when reading longer texts, while there was no significant difference when reading short texts [41].

These studies indicate that even though annotated reading shows great potential, divergent results may arise due to different reading medium, research participants, instructional design or even test instruments. Hence the effectiveness of our blended instructional design on improving learners’ reading competence remains to be verified through practice. The results can contribute to the accumulation of empirical evidence supporting annotated reading.

In addition, existing studies on annotated reading tend to generalize findings across all learner groups [22–24], overlooking within-group or individual variations. Regarding whether annotated reading leads to varying performance among participants, to our best knowledge, only Yeh, Hung, and Chiang [27] discussed intra-group differences.

Their research adopted RT as instructional framework and recruited 54 EFL learners at a Taiwan university to conduct online annotated reading. The results revealed that participants’ reading comprehension was enhanced after the intervention, but there were groups making more progress and less progress. With an analysis of two sample students’ reading process, the student making more progress “not only frequently reviewed their previously generated predictions, clarifications, questions, and summaries but also actively provided feedback to their peers in a reciprocal manner” (P1). This study shifted our attention from outcomes to processes, which is conducive to unveil “the black box” of annotated reading process.

However, their study’s reliance on a single class for pre- and post-tests and its focus solely on online learning processes may limit the generalizability and robustness of the findings. To bridge the gap, we adopted a between-subjects and within-subjects design. This approach allows for more effective control of variables and ensures the reliability of the results. Moreover, while the existent study focused mainly on students’ online performance, our instructional design consisted of four stages, enabling us to examine students’ performance at each intervention stage for a holistic view of their learning process. Additionally, compared with only one representative student from each group as a case sample [27], more students were recruited in our study to have interviews so as to get a nuanced picture of their perceptions and actualization of their reading practices.

### 2.3. The present study

Given the drawbacks and benefits of existent annotated reading methods, we designed a four-stage blended annotated reading framework to provide more affordances for EFL learners. However, its effectiveness to improve students’ reading competence needs empirical evidence. As students are distinctive individuals, one instructional intervention may yield different outcomes. Thus, whether intra-group differences in reading performance exist is another question worthy of investigation. Exploring students’ perceptions and actualization of this framework would contribute to the pedagogical development of English reading research. Therefore, this study intended to address the following research questions:

- Does the blended instructional design of annotated reading have an impact on students’ reading competence across the semester?
- What are students’ perceptions of and actual engagement in this annotated reading practice?

## 3. Method

### 3.1. Participants and context

This quasi-experimental study involved 108 second-year non-English major undergraduates (aged between 18–22) in a south China university, with 54 in the experimental class (EC) and 54 in the control class (CC) under the instruction of the same teacher. To contextualize the scale and significance of English language learning in China, it is noteworthy that the country had already surpassed 390 million English learners and users by 2000 [42], and subsequent research (e.g.[43],) indicates continued growth in the English learning population. However, despite over ten years of English instruction, the participants in this study exhibited lower to intermediate proficiency, with nearly half having only just passed the College English Test Band 4 (CET4, approximately equivalent to A2 on the CEFR). This proficiency profile is common among a substantial segment of English learners in China, highlighting the practical relevance of this study for understanding instructional interventions in large-scale, compulsory English education contexts. Such contextualization enhances the transferability of the findings to similar educational settings, both within and beyond China.

The course under investigation was *College English III*, which was also the only English-related course they had across the semester. This course was 80 minutes long per week, comprised of English reading, listening, speaking and writing, and annotated reading mainly took place in the reading part. With a few students’ major switch and absence on the tests, 42 students in the CC and 51 in the EC attended the whole learning process.

This study was approved by the Ethics Approval Committee of the first author’s university and the recruitment period was from 2/9/2024–31/12/2024. Oral consent was obtained in accordance with the procedures approved by the same Ethics Approval Committee. The session was witnessed by a colleague from the first author’s university who was neither involved in the research project nor had any prior or ongoing interaction with the student participants. This ensured there was no conflict of interest or power dynamic that could influence the participants’ willingness to consent freely. The first author briefly introduced the research objective and protocol, and then read a script, accurately translated into Chinese, aloud to the participants. This was to ensure they understood that there were no foreseeable significant risks associated with their participation and that they could withdraw from the study at any time without any negative consequences. It was also stressed that all information they provided would be kept strictly confidential. Subsequently, students were allowed to ask questions, and the first author answered them all to their satisfaction. Following this, all participants verbally indicated their understanding and agreement to participate in the study. The witness observed the entire process and signed a Verbal Informed Consent Witness Record, providing her full name, telephone number, and email address. This record has been retained by the research team.

After the quasi-experiment concluded at the end of the semester, six student participants were selected for semi-structured interviews. These interviews aimed to explore their nuanced perceptions of the proposed framework and document their authentic learning processes. Prior to every interview session, researchers outlined the study’s purpose, assured them of complete data confidentiality, and clarified that they retained the right to discontinue participation at any stage without penalty. All interviewees signed a formal written informed consent form before their interviews.

Furthermore, all experimental and interview procedures were conducted in strict accordance with standard research integrity guidelines. Rigorous safeguards were put in place to mitigate any potential harms to individual participants, relevant institutional bodies, and the wider scientific enterprise [44].

### 3.2. The digital annotation tool: Tencent Document

Tencent Document is a widely used document collaboration platform in China that allows (a)synchronously real-time online editing by multiple users. It is free of charge and convenient for users to access and edit documents on different devices such as computers, mobile-phones or tablets. In this research, students can annotate certain parts of an English text, add website links, post pictures or videos, read and respond to others’ annotations, which offer opportunities for students to express thoughts, observe peer performance and have interaction in an online setting. It also opens a window for the teacher to assess what students have or haven’t known about the text and thus provide targeted guidance.

### 3.3. Research design

The instructional design contains four stages (see Table 1), commencing with individual paper-based annotated reading, followed by a collaborative phase where group members share, assess, discuss, and integrate their annotations based on assigned annotation types. The nine annotation types employed (see Table 2) were adapted from widely used Chinese annotation practices. Due to the large number of groups and limited class time, annotation types were assigned on a rotating basis to ensure students focused on different skills in each session. Following group discussions, one member was designated to post the collaborative results to Tencent Document. Meanwhile, all other members were required to freely contribute by creating or responding to at least one additional annotation based on their own understanding. The final stage was a in-class phase taking 80 minutes for each text. A representative from each group reported their findings, after which the teacher provided feedback and encouraged peer interaction. Subsequently, students were expected to reflect on the entire process and recorded new annotations from the reports into their textbooks. These activities aimed to cultivate a deep, dialogical engagement with the texts and the learning community, guiding students through a structured process of reading, thinking, writing, discussing, and reflecting.

**Table 1:** The four-stage annotated reading activities.

	Activities	Phases
Stage 1	Paper-based annotated reading	Individual text reading
		Make annotations on paper textbook
Stage 2	Group discussion	Discuss annotations on the given type
Stage 3	Social annotated reading	Submit group discussion results to Tencent Document
		Post/Respond to at least one individual online annotation
Stage 4	Class presentation, feedback and reflection	Group presentation
		Teacher/Peer feedback
		Students’ reflection

**Table 2:** Annotation types: methods & purposes.

Annotation Type	Method	Purpose
Basic Annotation	- Mark unfamiliar words/phrases with pronunciation or definitions/paraphrases - Highlight/underline key sentences	To aid preliminary understanding and identify core ideas
Reflective Annotation	- Record personal interpretations, emotions, or confusions - Include external insights	To enhance comprehension and uncover author’s intent
Evaluative Annotation	- Provide evaluative comments on content/ language/ style - Offer praise or critique	To foster critical thinking
Associative Annotation	- Note connections to related texts, historical figures, or events	To bridge concepts across contexts
Method-based Annotation	- Identify rhetorical devices/writing techniques - Analyze their effectiveness	To improve language application skills
Appreciative Annotation	- Select favorite paragraphs/sentences - Write analytical commentary	To explore literary merits
Questioning Annotation	- Jot down questions that arise during reading	To stimulate critical thinking and further inquiry
Supplementary Annotation	- Fill in missing background information - Clarify ambiguities	To provide comprehensive understanding
Imitative Annotation	- Select well-crafted lines - Write sentences mimicking their style/structure	To internalize effective writing techniques through active imitation

This research lasted sixteen weeks. In the first week, the teacher introduced annotated reading, annotation types, the use of Tencent Document and the research purpose to the EC. All the students agreed to participate, took the pre-test and formed groups of 4–5 for later annotating activities. Four in-class presentation sessions were held in Weeks 3, 6, 10, and 13 for students to report on their annotated texts. The post-test was held in Week 16, with interviews conducted within one week after the test results were released.

As for the CC, they were identical to the EC in all respects (same teacher, same materials, same course structure) except that during the four target weeks, they received traditional lecture-based instruction on the target text while the EC engaged in class reports and feedback focusing on annotations. The pre-test and post-test for the EC were conducted on the same days and used the same papers as those for the CC.

### 3.4. Data collection and analysis

The data collection and analysis consisted of two phases.

First, two reading tests were conducted in the first and sixteenth weeks of the semester for the CC and the EC, serving as pre-test and post-test respectively. Each test used four CET-4 reading passages (300–400 words each, with five multiple-choice questions per passage) and lasted 40 minutes.

SPSS 29 was used to analyze quantitative data. Initially, an independent samples *t*-test assessed EC-CC differences at pre-test and post-test, while a paired samples *t*-test checked pre-test-post-test differences within the EC and CC. For the EC, individual gain score analysis was further performed, with graphical representation of each participant’s pre-test-post-test differences.

In the second phase, based on the learning gains within the EC, six students (three with the highest and three with the lowest gains; see Table 3) were purposefully selected for semi-structured interviews. The interview protocol (see data in S1 Appendix) comprised eight main questions regarding their annotated reading process, perceptions and actualization. All interviews, conducted in Mandarin to ensure comfort and fluency, lasted 15–25 minutes via the video-conferencing platform Tencent Meeting. The recordings (totaling 119 minutes) were transcribed, yielding 31,959 Chinese characters, which were member-checked by participants. Any unclear points were subsequently clarified through follow-up chats via WeChat, a Chinese social media app.

**Table 3:** Interview participants.

Interviewees (pseudonyms)	Gender	Age	Gains in reading tests
Lily	female	20	+60
Emily	female	20	+50
Rose	female	20	+40
Jack	male	19	−35
Kate	female	19	−25
George	male	20	−25

A thematic analysis approach was employed to compare how two groups with distinct learning outcomes perceived and practiced the four-stage annotated reading. We followed a structured six-step process to ensure systematic coding, theme identification, and cross-group comparison [45].

The analysis started with the three interviews from the group with most gains. Using NVivo 19, the authors repeatedly reviewed the transcripts for familiarity, segmenting the texts into meaning units. Through iterative open coding, preliminary concepts (e.g., “gain a more comprehensive understanding of the text”) were generated, which were subsequently grouped via axial coding into broader themes (e.g., “text-based benefits”). These themes were refined and organized within the four-stage instructional framework, alongside salient categories like “perceived benefits”. The same analytical procedure was then applied to the interviews from the group of least gains, followed by a comparative analysis to identify convergences and divergences. To enhance credibility, representative direct quotations were selected to substantiate the themes [46]. The authors collaboratively conducted all analyses, resolving disagreements through discussion until consensus was achieved.

## 4. Results

### 4.1. Change in students’ reading competence between the EC and the CC

Prior to conducting the paired-samples and independent-samples *t*-tests, the normality of the pre-test and post-test data for both the CC and EC was examined. As shown in Table 4, the Sig. for pre-test and post-test results of the CC was 0.45 and 0.15 (≥0.05) respectively, indicating that they were normally distributed. However, the results for the EC indicated a slight deviation from normality.

**Table 4:** Normality of pre-test and post-test data.

	Kolmogorov - Smirnov^a^			Shapiro – Wilk		
Group	Statistic	df	Sig.	Statistic	df	Sig.
Pre-test for CC	.10	42	.20*	.97	42	.45
Pre-test for EC	.13	51	.04	.98	51	.54
Post-test for CC	.10	42	.20*	.96	42	.15
Post-test for EC	.13	51	.03	.97	51	.30

*. This is a lower bound of the true significance.

a. Lilliefors Significance Correction

Thus, a Mann-Whitney U test (Table 5) was used to compare the scores between the CC and the EC, U = 1,042.50, *Z* = −0.22, *p* = 0.83 (>0.05), *r* = 0.02 in the pre-test. This indicates no statistically significant difference in pre-test scores between the two classes, confirming their comparability prior to the intervention.

**Table 5:** Mann-Whitney U test results comparing pre-test results between the CC and EC.

statistic	numerical value
Mann-Whitney U	1,042.50
Wilcoxon W	1,945.50
Z-score	−0.22
Asymptotic Significance (Two – tailed)	*p* = 0.83
Effect size (r)	*r* = 0.02

a. Grouping variable: Class

The Mann-Whitney U test showed a statistically non-significant post-test difference between the EC and CC (*p* = 0.32, see Table 6). However, the effect size rose from *r* = 0.02 at pre-test to *r* = 0.10 at post-test, which is considered a small effect according to Cohen’s [47] conventions. This upward trend indicates that the instructional intervention produced some measurable practical impact. In addition, a visual comparison of the average scores (see Fig 1) confirms this pattern: from similar starting points, the EC’s average post-test score rose more substantially than the CC’s, suggesting a stronger intervention effect.

**Table 6:** Mann-Whitney U test results comparing post-test results between the CC and the EC.

statistic	numerical value
Mann-Whitney U	942.50
Wilcoxon W	1845.50
*Z*-score	−1.00
Asymptotic Significance (Two – tailed)	*p* = 0.32
Effect size (r)	*r* = 0.10

a. Grouping variable: Class

**Fig 1:**
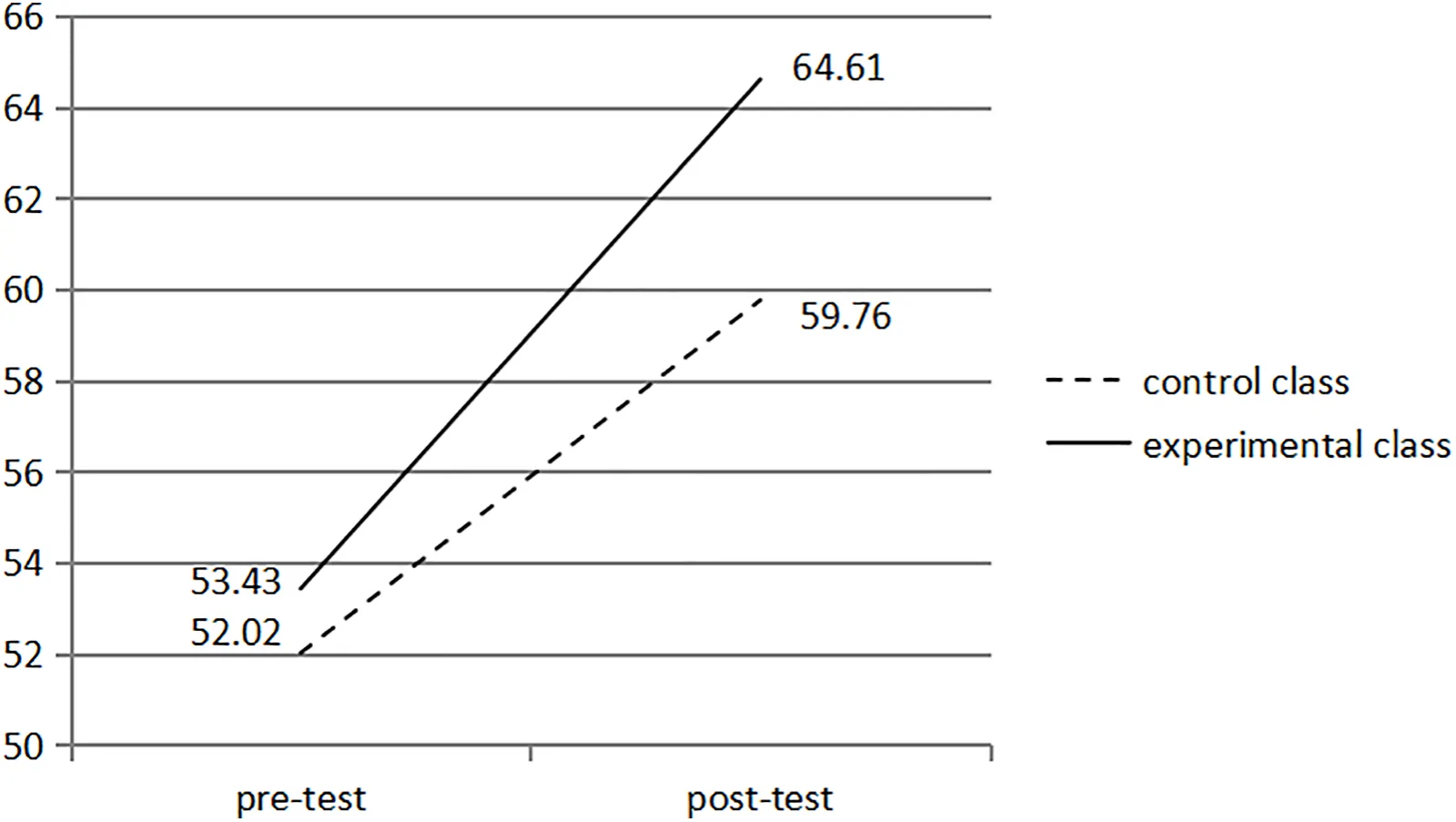
The change of average scores between the pre-tests and post-tests. A line chart comparing the average reading test scores of the EC and the CC, showing a greater score increase from pre-test to post-test for the EC. The EC’s pre-test average was 53.43 and its post-test average was 64.61, while the CC’s were 52.02 and 59.76, respectively.

Beyond cross-group post-test comparisons, within-group score changes over the semester were further explored via paired-samples analyses. For the CC, a paired-samples *t*-test was performed owing to its normally distributed data.

The paired-samples *t*-test (see Table 7) revealed a statistically significant difference between pre-test and post-test scores for the control class (*n* = 42), *t* = −2.37, *p* = .023, Cohen’s *d* = −0.37, 95% CI [−0.68, −0.05]. The negative effect size indicates that post-test scores were higher than pre-test scores, with a small-to- medium practical effect.

**Table 7:** Paired-samples *t*-test results for pre-test and post-test scores in the CC.

Pair	Variable	Mean Difference	Std. Deviation	Std. Error Mean	95% CI for Difference	*t*	df	*p* (2-tailed)	Cohen’s *d*	95% CI for *d*
1	Pre-test vs. Post-test (CC)	−7.74	21.19	3.27	[-14.34, -1.14]	−2.37	41	.023	−0.37	[-0.68, -0.05]

In light of its slight deviation from normality, the EC’s pre-test and post-test scores were compared using the Wilcoxon signed-rank test. The results (see Table 8) showed a statistically significant improvement in post-test scores compared to pre-test scores, *Z* = −3.844, *p* < .001. The effect size was calculated as *r* = .54, indicating a large effect. This finding demonstrates that the instructional intervention had a strong positive impact on students’ performance.

**Table 8:** Wilcoxon signed-rank test results for pre-test and post-test scores in the EC.

Variable Pair	*n*	Negative Ranks (*n*, Mean Rank, Sum of Ranks)	Positive Ranks (*n*, Mean Rank, Sum of Ranks)	Ties (*n*)	*p*	Effect Size (*r*)	95% CI for *r*
Pre-test vs. Post-test	51	7, 27.14, 190.00	39, 22.85, 891.00	5	<.001	.54	[.30,.70]

Although both groups achieved statistically significant within-group improvements in reading performance, the discrepancy in effect sizes was notable: the EC showed a large effect (*r* = .54), whereas the CC only had a small-to-medium effect (Cohen’s *d*=−0.37). This indicates that the instructional intervention yielded a substantial practical effect on the experimental class, even though the between-group post-test difference did not reach statistical significance.

To examine individual differences within the EC, we analyzed the change of each student’s pre-test to post-test score. Results were mixed (see Fig 2): 39 students improved, 7 declined, and 5 showed no change. The gains among improving students were substantial (up to 60 points), while some declines were also notable (up to −35 points). Based on this distribution, students were categorized into a group of gain (GG) for those who improved and a group of decline (GD) for those whose scores decreased.

**Fig 2:**
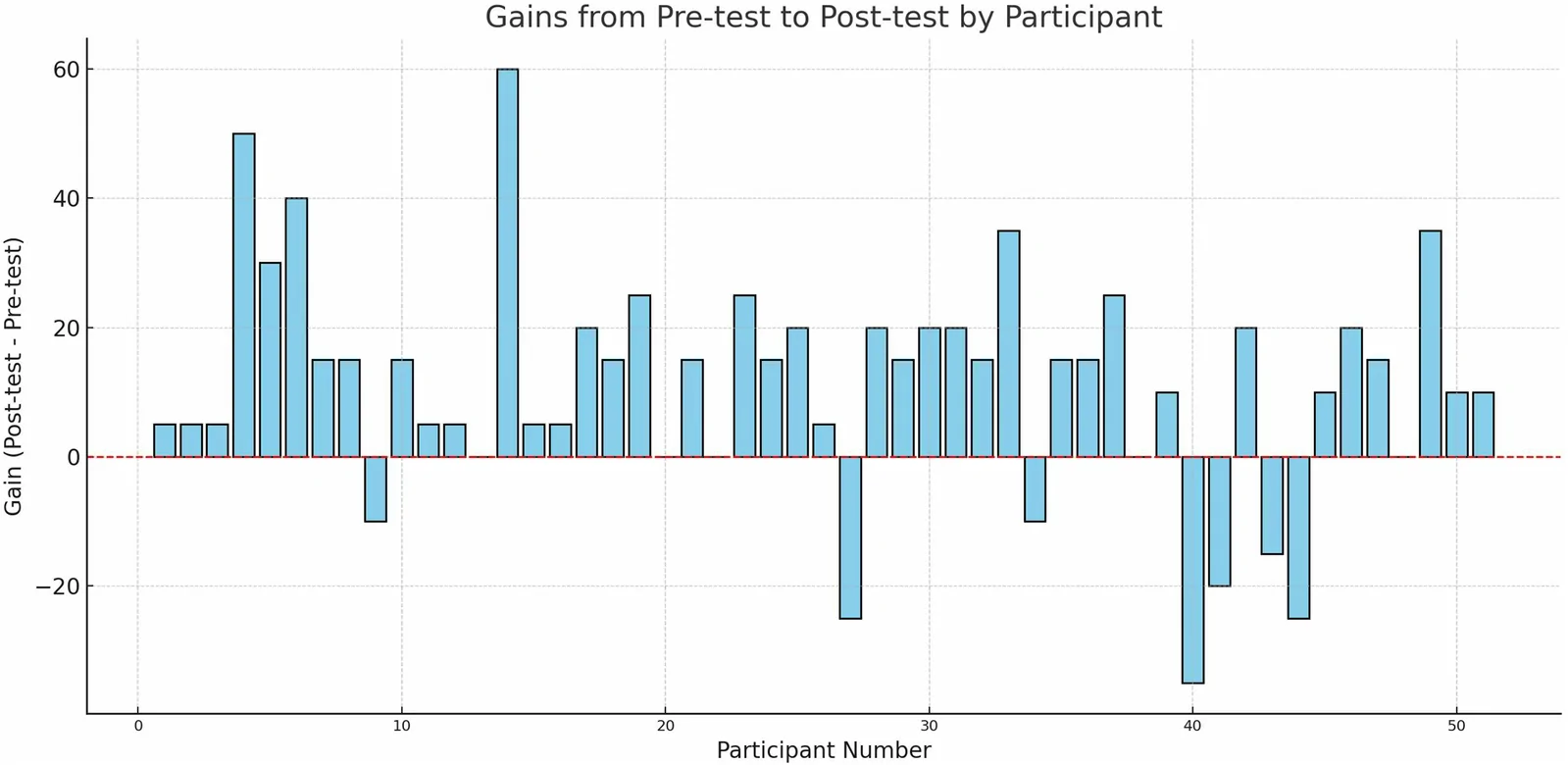
Gains from pre-test to post-test by participant in the EC. A bar chart displaying the reading score improvement for each individual student in the EC, showing that 39 students made a positive gain, 7 declined, and 5 showed no change.

### 4.2. Students’ perceptions and actualization of the annotated reading practices

The qualitative analysis revealed that all interviewees benefited from the annotated reading practices but also encountered challenges during the process, with the GD reporting notably more difficulties. Furthermore, considerable differences emerged in how students implemented the first three stages of the instructional design.

#### 4.2.1. Students’ perceptions of the annotated reading practices.

Students from both groups reported a range of benefits from the instructional design. As shown in Table 9, the distribution of qualitative codes was broadly similar across groups, suggesting that the instructional framework supported learning progress among all participants. The most frequently reported category was “*Enhanced Cognition and Skills”*, which reflected deepened text comprehension, improved English proficiency, and strengthened reading skills. In the category “*Increased Motivation and Shifted Attitudes*”, students described greater learning autonomy and a transition from initial anxiety to increased comfort with the learning process. “*Development of Metacognition and Self-Directed Learning”* captured students’ efforts to optimize reading workflows, refine collaborative strategies, and enhance information retrieval skills. Finally, “*Enhanced Application and Transfer Awareness*”, though the least coded category, indicated that some students began to apply annotated reading strategies to other contexts, including exam situations.

**Table 9:** Students’ perceived benefits from the GG and the GD.

Category	Number of codes (GG)	Number of codes (GD)	Sample
Enhanced Cognition and Skills	20	16	In Unit 1, we were required to do associative annotation. I searched for relevant concepts (of species) beforehand and developed a profound comprehension of the subject. (Kate)
Increased Motivation and Shifted Attitudes	6	6	My autonomy in learning has greatly improved. Last semester, that teacher didn’t require us to do this, so I basically never even looked at the textbook…Now the progress is that I force myself to read. I have to think about it, read it twice and think.(Lily)
Development of Metacognition and Self-Directed Learning	7	6	Generally, I usually read through it first. Then, on the second reading, I would deliberately look for parts that our team was assigned to annotate. I’d also search online (for relevant information) at the same time.… If I can’t find enough points, I would read it again for a final check. (Emily)
Enhanced Application and Transfer Awareness	3	2	I think there is indeed an effect (for CET-4 preparation). Without it, I’d probably do very little (English) reading on my own. （Rose）

It is noteworthy that the codes for “*Enhanced Cognition and Skills*” were disproportionately concentrated compared to those for “*Enhanced Application and Transfer Awareness*”. This suggests a potential misalignment between the students’ perceived gains in language and skills and their awareness of how to strategically transfer these competencies to new contexts.

Interviewees identified three categories of difficulties in conducting annotated reading. Notably, they reported far fewer difficulties compared with the perceived benefits. As Table 10 indicated, students in GD obviously experienced more difficulties in the learning process.

**Table 10:** Students’ perceived difficulties from the GG and the GD.

Category	Number of codes (GG)	Number of codes (GD)	Sample
Pedagogy-based Difficulties	2	4	The language in Unit 6 was cold and formal. …We wondered if the text wasn’t suitable for evaluative annotation. (Kate)
Individual-specific Obstacles	1	3	Because my English might not be very good, I needed to use online learning platforms to assist my annotations. (George)
Equipment-related Problems	1	3	I used my phone to log in it. Maybe there is a problem with my phone – it lagged a bit. (Jack)

The first category is “*Pedagogy-based Difficulties*”. For all the interviewees, this was their first exposure to English annotated reading and such detailed classification of annotation types. Although the teacher provided examples, students reported their unfamiliarity with certain annotation types, puzzle on how to conduct annotated reading, and concern over time-consumption. Next, “*Individual-specific Obstacles”* mainly referred to the mismatch between students’ English proficiency and the reading materials as well as their incapability to understand western culture. The third is related to equipment, covering both hardware and software.

#### 4.2.2. Students’ actual engagement in the annotated reading practices.

While all interviewees reported completing the annotated reading tasks as designed, students in the GG exhibited significantly different engagement patterns from those in the GD across paper-based annotation, group discussion and SA on Tencent Document.

For paper-based annotated reading, interviewees from the GG usually read a text two or more times to get prepared for group discussion, while those in the GD at most read it twice to do the same task. For example, Rose recounted her working procedure, “*I prefer to read through the text first to get a general understanding of its meaning, then divide it into sections based on paragraph structure. After that, I will read it carefully in the third round.*”

Emily followed a similar process but more focused on the assigned annotation type.

*“I usually read through it first. Then, on the second reading, I will deliberately look for parts that our team was assigned to annotate. I will also search online (for relevant information) at the same time. … If I can’t find enough points, I will read it again for a final check.” (Emily)*

Compared with their peers who engaged in meticulous paper-based annotation, students in the GD invested noticeably less time and effort in this stage. As Jack noted, “*I could do it in one go. The texts seem relatively simple.*” In contrast, Kate and George found the texts challenging and resorted to alternative strategies for comprehension. Kate used WELearn, a textbook companion app offering Chinese translations, to grasp the main ideas quickly. Similarly, George turned to online tools like English-Chinese dictionaries. In both cases, these external resources significantly reduced their text preparation time.

Group discussion is supposed to be an important stage to collaborate with team members to pool ideas, evaluate opinions and make decisions before class presentation. Although we preferred students to have face-to-face discussions, interviews showed that the two groups experienced markedly different modes, atmospheres and procedures.

As for modes of discussion, in the GG, Lily’s group mainly discussed online, Rose’s group had an alternation of online and offline discussion to cater to group members’ needs, and Emily’s group preferred to talk face to face but they also had a WeChat group to share materials. However, members in the GD all adopted face-to-face discussion.

Regarding the discussion atmosphere, students in the GG described their interactions as “*democratic”* (Rose) and “*quite amicably”* (Lily). Emily’s account of their collaborative procedure was representative, “*After paper-based annotation, we would report our findings for collective review. When information was abundant, we discussed which parts were the most valuable and refined the content to make it more concise*.”

In sharp contrast, students in the GD were less productive, largely due to a lack of strong leadership and a collaborative learning atmosphere. When reflecting on their discussion, George criticized the group leader for having “*low presence*”—acting more like an ordinary member than a leader. He also noted that some members were disengaged, while others communicated insensitively, triggering unnecessary disputes. This ineffective collaboration dampened George’s emotional investment, leaving him reluctant to engage in future group work.

Unlike George’s group, Jack’s team collaborated like a jigsaw puzzle without real discussion. They divided the annotation task by paragraph, created individual slides, and delegated consolidation and presentation to the member with the smallest share—merely stitching together separate contributions.

The social annotated reading session on Tencent Document was designed to broaden students’ horizons and foster a dynamic exchange of ideas through collaborative annotation. However, the two groups approached it quite differently. In the GD, Jack and Kate did not read online annotations at all. Kate candidly attributed this to technical limitations and a lack of self-discipline—while she preferred using her phone, software issues prevented her from viewing annotations on it. The inconvenience of having to retrieve her computer to read them often deterred her from making the effort. Jack cited both physical and practical reasons. Reading long sentences on his phone caused discomfort, and more importantly, he felt the annotations from other groups were largely irrelevant to his own group’s assigned task, which diminished his motivation to read them.

Compared with their inactivity, students in the GG invested more effort on it. All three participants reported reading online annotations though with different approaches. Lily mentioned she “*would read them all …not super carefully.*” Rose was more task-orientated, “*If my group was assigned a specific task, I read every comment carefully. Otherwise, I’d just scan to see if others’ findings aligned with mine.*” Similarly, Emily showed particular interest in annotations related to her own, “*I paid special attention to comments in the sections I had annotated, curious to see what others had to say.*”

The final stage involved class presentations, feedback and reflection. All participants reported listening to presentations, though they also admitted focusing more on engaging content while paying less attention to the boring segments. While some students took notes, others did not. Overall, at this phase, students in the GG exhibited no significant behavioral differences from those in the GD.

## 5. Discussion

This research proposed a four-stage annotated reading framework and employed a quasi-experimental design to evaluate its effectiveness in enhancing students’ reading competence. In addition to collecting and analyzing quantitative data to measure changes in participants’ reading scores, semi-structured interviews were conducted to gain a nuanced understanding of students’ perceptions and actualization of their reading practices.

Aligned with the findings by Johnson et al. [22], and Yang and Hu [40], our research showed that there was no statistically significant difference between the CC and EC in pre-test and post-test scores. One possible reason may lie in the short timespan and limited annotated reading practices for the intervention -- across the semester only 4 weeks was devoted to annotated reading of 4 texts. Such a brief duration and limited number of reading texts appeared insufficient for students to fully internalize this innovative reading method and transfer it to practical reading tasks. This was further reflected in their weak perception of “*Enhanced Application and Transfer Awareness*”(see Table 9). Therefore, we fully support researchers’ calling for longer intervention and observation [13,16,48,49]. The other reason might be due to the number of students in the EC and the teacher’s workload. In this research, there were 54 students in the EC and the instructor had 6 classes with similar sizes which made it difficult for her to pay attention to students’ individual performance. Without the teacher’s monitoring, those in need of “*other-discipline*”(Rose) may not make full use of the instructional scaffolding. Despite the statistically insignificant difference between the post-test scores, it is worthy to notice that the gap between the two classes was widening and students in the EC demonstrated stronger potential in reading performance than their peers. Given longer intervention and more reading practices, it is plausible that future research will report positive findings [50].

As for the score changes within the EC, divergent learning outcomes emerged with some students gaining progress, some staying unchanged, while others declined. This partially aligns with Yeh, Hung, and Chiang [27] who also reported intra-group differences -- all students improved, albeit to varying degrees. The difference between their results and ours may stem from complicated individual differences in learning motivation, habits, engagement, etc. But it is clear that students who benefited from this pedagogy exhibited greater learning engagement than those experiencing decline. Interviews revealed, students in the GG surpassed those in the GD in frequency of text reading, quality of group discussions, and agency in reading online annotations. Unlike prior research [13,14] which reports overall engagement without addressing individual variation, the current study reveals substantial differences among students within the same framework. This suggests that the pedagogy’s efficacy largely depends on students’ willingness to invest ample time, effort, and affect.

As reading competence was generally recognized as “a multifaceted skill” [33], interviewees perceived a range of benefits (see Table 9), which supported nearly all the core concepts of reading competence [32,34–37]. To be more specific, compared with the existent mainstream research mainly adopting social annotated reading [13,14,26,27], our instructional design provided a structured and all-rounded scaffolding that enabled students to take in affordances from different learning medium by increasing preview time, promoting text comprehension, facilitating interactive learning and improving reading and critical thinking skills.

However, the perceived “*Enhanced Cognition and Skills*” (36 codes) outnumbered “*Enhanced Application and Transfer Awareness*” (5 codes), indicating that students effectively acquired knowledge and skills from the framework, yet they encountered difficulties when applying them to other contexts. This may be related to their prior exam-oriented learning model [51] and limited access to English reading materials. Therefore, teachers should provide targeted guidance and detailed examples to fill this gap.

One inconsistency with previous research is also worth discussion. In our study, three interviewees from the GG respectively adopted online, face-to-face or hybrid discussions without compromising the efficiency and quality of their group work. Conversely, despite the exclusive use of face-to-face discussion in the GD, this mode yielded limited benefits because George’s group leader was irresponsible and members’ passivity undermined the discussion atmosphere, while Jack’s group adopted a jigsaw-style cooperation failing to foster productive intellectual exchange. These findings are different from those [19,20] who reported face-to-face discussion was more effective. Thus, we argue that modes of discussion are not the decisive factors in shaping learning outcomes; rather, the quality of group dynamics and the depth of student engagement are equally critical.

In this study, students perceived three categories of challenges hindering the effective adoption of the pedagogical design (see Table 10). To our knowledge, pedagogy-based difficulties have not yet been addressed by prior research because existing studies mainly focus on basic annotation strategies without intricate instructional design [22–24]. Our framework incorporated a wider variety of annotation types, aiming to provide students with enhanced scaffolding. Individual-specific challenges mainly refer to the mismatch between students’ English proficiency and text difficulty, which aligns with the findings that English proficiency mediates instructional effectiveness [28,52]. Regarding equipment-related problems, our finding echos reports by Kawase et al. [18] that SA tools and hardware imposed burdens on students. For these difficulties, we proposed suggestions in the next part. But it is also worth noticing that students in the GG reported much fewer challenges than those in the GD, which again supports that students’ engagement and motivation are vital factors for academic success.

In summary, this study demonstrates that the four-stage annotated reading instructional design has potential in improving students’ reading competence, and that students perceived substantially more benefits than difficulties in the learning process. These findings confirm the effectiveness of annotated reading [7,8,12]. The intra-group differences in the EC indicated those who invested more time, effort, and affect reaped more. Hence, we argue that students’ engagement should be prioritized by instructors.

## 6. Conclusions and implications

The instructional framework introduced in this study moves beyond the prevalent approach of relying exclusively on online SA. By addressing the complete learning process, it aligns more closely with the authentic needs and contexts of EFL instruction. Although the quantitative results did not reach statistical significance, the qualitative data suggested students’ development across multiple sub-dimensions of reading competence. Furthermore, the findings reveal a meaningful pattern that modes of learning (e.g., online vs face-to-face discussion) are not decisive factors in shaping learning outcomes. Instead, students’ engagement appears to play a pivotal role (S1 Table). Therefore, in addition to meticulous instructional scaffolding, educators should prioritize the cultivation of students’ intrinsic motivation to enhance their engagement and performance.

To maximize student learning, the following suggestions are proposed to address the difficulties perceived by students. Firstly, sufficient training should be provided at the outset to help students master various annotation types and techniques. Secondly, teachers should be aware of students’ varying English proficiency and tailor reading materials to maximize educational outcomes [24] for if the texts are too easy, students may not dive deeper into the content; if too difficult, they may be intimidated into taking shortcuts. For students with lower English proficiency, teachers should impart reading strategies (e.g., inferring meaning from context) and foster active discussions to resolve challenges so as to reduce overreliance on translation tools. Lastly, reliable annotation tools should be selected and technical support from the institution sought to minimize hardware or software burdens. But most importantly, students should be encouraged to take greater initiative in English learning.

Despite its potential contributions, this study has several limitations. Although this study spanned 16 weeks, the actual teaching intervention lasted only four weeks and involved four texts. To more thoroughly evaluate the efficacy of the four-stage annotated reading framework, it is necessary to validate it over a longer time frame (such as one academic year). Additionally, even though the unequal attrition (42 in the CC vs 51 in the EC) was largely exogenous, the imbalance may still introduce potential bias. To mitigate this concern, future studies should carefully estimate expected attrition rates a priori and recruit a larger initial sample accordingly. Furthermore, since reading competence is a multifaceted construct, this study’s reliance on pre- and post-test scores limited its scope to overall proficiency gains, leaving the framework’s impact on other dimensions without being fully explored. Future research could address this by investigating areas such as the emotional responses elicited during the intervention.

## Supporting information

S1 Table Complete quantitative dataset (Excel file). (XLSX)

S1 Text Anonymized interview transcripts (English version). (DOCX)

S1 Appendix Major questions for semi-structured interviews. (DOCX)
