# Supplementary material for: Developing students’ reading competence in an EFL context through annotated reading: A blended instructional design
Source: PLoS One. 2026 Oct 14;21(10):e0357895. doi: 10.1371/journal.pone.0357895 (PMC13667772; doi:10.1371/journal.pone.0357895)
Supplement: Supplementary file 1 [file pone.0357895.s004.pdf]

This manuscript addresses a timely and pedagogically relevant topic by examining whether a blended annotated reading design can support the development of EFL students' reading competence. The attempt to move beyond purely online annotation and to incorporate students' perceptions and actualization is valuable. The study also has the potential to contribute to ongoing discussions about technology use, learner engagement, and the broader teaching-learning process. However, the current version still requires several revisions before publication can be considered.

Comment 1:

Your manuscript is situated within the Chinese EFL context; however, the broader significance and scale of this setting are not yet immediately clear to international readers. At present, the manuscript provides some useful local contextual information. For example, the participants are described as "**108 second-year non-English major undergraduates**" in "**a south China university**" taking **College English III**, and their English proficiency is briefly characterized through **CET-4**. While these details are helpful, they mainly describe the immediate classroom setting **rather than the wider Chinese EFL landscape**. I therefore suggest strengthening the Method or Introduction section by adding a concise overview of the scale and significance of English language learning in China. This will help readers better appreciate both the broader relevance and the transferability of your study.

For example, you can write something like: "China had already surpassed 390 million English learners/users by 2000 (Wei & Su, 2012), and subsequent work (e.g. Wei & Gao, 2022) suggests the continued growth of English learning in China." Such contextualization would make the study's contribution more accessible and meaningful to an international readership.

References:

Wei, R. & Gao, X. (2022). New research trends in TESOL: An introduction. *Chinese*

Journal of Applied Linguistics, 45(2), 220-223.

Wei, R. & Su, J. (2012). The statistics of English in China: An analysis of the best available data from government sources. *English Today, 28* (3), 10-14.

Comment 2:

You are requested to consistently use the term ‘statistically significant’ instead of ‘significant’. The omission of the term ‘statistically’ can easily result in the erroneous impression that the results are important. Hence many scholars (e.g. Carver, 1993) have underlined that the term ‘statistically’ must always precede the word ‘significant’.

References:

Carver, R. P. (1993). The case against statistical significance testing, revisited. *The Journal of Experimental Education, 61*(4), 287–292.

Comment 3:

Echoing Comment 2 about the over-reliance on the statistical significance level (viz. p), this comment addresses the same issue in your manuscript regarding the use of **effect sizes**. In the quantitative section, the manuscript reports Mann-Whitney U test results mainly in terms of p values (e.g., **$p = .83$ and $p = .32$** in Line 320 and Line 327), but **no corresponding effect sizes are provided.** This is not sufficient. Best reporting practice in applied linguistics and beyond requires researchers to report effect sizes for **both statistically significant and non-significant results** (Larson-Hall & Plonsky, 2015; Wei et al., 2024). In other words, p values alone do not tell readers how large, small, or practically meaningful the observed differences are. Effect sizes are therefore indispensable, as they provide crucial information about the magnitude of the observed effect and facilitate more meaningful interpretation and comparison across studies.

I strongly recommend that you adequately report and interpret effect sizes throughout

the quantitative section. For the Mann–Whitney *U* tests, an *r*-family effect size can be reported; for any *t* tests used, Cohen's *d* should also be reported and interpreted based **on an explicit benchmark system**. At present, the manuscript's statistical interpretation remains overly *p*-value-driven.

If you need good examples of the reporting and interpretation of effect sizes, please consult Wang & Wei (2024) and Shen and Wei (2025).

References:

Larson-Hall, J., & Plonsky, L. (2015). Reporting and interpreting quantitative research findings: What gets reported and recommendations for the field. *Language Learning, 65*(S1), 127–159.

Shen, J., & Wei, R. (2025). Is multilingualism linked to attitudes toward gender equality? Evidence from a big data survey. *Journal of Multilingual and Multicultural Development*. Advance online publication. https://doi.org/10.1080/01434632.2025.2552327

Wang, J., & Wei, R. (2024). Is bilingualism linked to well-being? Evidence from a big-data survey. *Bilingualism: Language and Cognition, 27*, 546–556. https://doi.org/10.1017/S1366728923000603

Wei, R., Reynolds, B. L., Kong, M., & Liu, Z. (2024). Is bilingualism linked to national identity? Evidence from a big data survey. *Journal of Multilingual and Multicultural Development, 45*(8), 3078–3092. https://doi.org/10.1080/01434632.2022.2085282

Comment 4:

The analysis for Fig. 5 (Line 339-344) appears to be a simple **individual gain-score analysis** based on each student's post-test minus pre-test score in the EC. In other words, the classification of "**39 students improved, 7 declined, and 5 showed no change**"

(Line 340) seems to be purely descriptive. However, although the Methods section states that “paired samples t-test checked pre-test-post-test differences within the EC and CC” (Line 278), the Results section does not appear to report those inferential results. Please clarify whether such within-group tests were actually conducted and, if so, report them fully. If the distributional assumptions were not met, a nonparametric within-group alternative may be more appropriate.

This issue should also be corrected in the revised manuscript. Importantly, the pre-test-post-test differences within the EC and CC are not a trivial detail, as they are necessary for understanding whether each group changed over time and for interpreting the intervention effect more rigorously in a quasi-experimental pre-test/post-test design.
