# Supplementary material for: Developing students’ reading competence in an EFL context through annotated reading: A blended instructional design
Source: PLoS One. 2026 Oct 14;21(10):e0357895. doi: 10.1371/journal.pone.0357895 (PMC13667772; doi:10.1371/journal.pone.0357895)
Supplement: Supplementary file 2 [file pone.0357895.s002.docx]

**S1 Text. Anonymized interview transcripts (English version)**

**Interview with Lily**

**Alice:** Don't be nervous, just think of it as the two of us having a casual chat.

**Lily:** We don't need to turn on video, right?

**Alice:** No, no need.

**Lily:** Okay, okay.

**Alice:** Can you see it over there? Have I started recording? Start voice recording. Okay, so the reason I asked to meet with you today is to talk about this annotated reading activity. Since I implemented it this semester, I wanted to get a sense of everyone's feelings about it. First, before we started this annotated reading activity this semester, had you encountered or tried this method before?

**Lily:** I think so, actually. In middle school and high school Chinese reading classes, teachers usually required us to read through the text first and make some annotations next to it. But it was quite free-form, without restricting us to a specific annotation method. It was more about circling any new vocabulary we didn't know or annotating our thoughts and feelings.

**Alice:** So it was relatively basic, right? Mainly new vocabulary. And primarily in the Chinese subject.

**Lily:** Yes. Yes, mainly Chinese.

**Alice:** There wasn't a mandatory requirement, right?

**Lily:** No, we hadn't tried it in English yet.

**Alice:** So you had some exposure to annotation in Chinese during primary and secondary school, but it was relatively superficial, mainly vocabulary and personal reactions. Not very in-depth or mandatory. So this semester when we did annotated reading, what did you learn from it?

**Lily:** I think compared to my English class last academic year, my autonomy in learning has greatly improved. Last semester, that teacher didn't require us to do this, so I basically never even looked at the textbook. I would just listen to him talk in class, occasionally take some notes. Sometimes I wouldn't even know where he was in the lesson and would have to search for the spot. Without annotated reading, I feel I hardly had any understanding of the textbook content. Basically, last semester, I just memorized the textbook vocabulary because there were dictations, and I took some notes during the teacher's lecture. But as for an overall understanding of the text or a deeper comprehension, there was none.

**Alice:** So before this semester, you followed the teacher's guidance, and your pre-class preparation wasn't sufficient, right? But through this method, you have to prepare yourself first. You understand the text content better. You can also keep up with the teacher's pace in class.

**Lily:** Exactly. Exactly.

**Alice:** Okay, aside from improving your autonomy, what about the content itself, like mastering the text or other aspects? Do you have any feelings or improvements?

**Lily:** Definitely. Because annotated reading requires me to have a certain overall understanding of the text. I need to read through it to grasp the main content, and then, based on the annotation type required – our group assigned Associative Annotation – I have to do some extra exploration based on its theme. For example, we needed to expand by introducing the author's writing background, the author's biography, interpretations of their other works, and making extensions on certain aspects of the text content. I think it's a process of deeply understanding the text. That's it.

**Alice:** Okay. So, we just talked about how, besides individual work, the groups had responsibilities for annotation tasks – we divided it into several stages. First, individuals annotate on the paper textbook, right? Then the group works on the task assigned by the teacher. When you do individual annotations, how many times do you usually read the text? Or do you just do it once?

**Lily:** I basically read it twice. Because the first time, there are many new words; I need to find them and grasp the general content. The annotation comes during the second reading, based on understanding the general content, then I think and annotate.

**Alice:** Okay. So first, you read the text individually, look up any words you don't understand, right? Get the general idea. Then the second time, you think about the part you need to annotate, explore it, and then write it down. Then, check it. Then during the group discussion stage, how did you do it?

**Lily:** We set up a group on WeChat, shared everyone's annotations, looked for any novel ideas, then summarized them for the presentation.

**Alice:** Did the group leader organize you, or did everyone speak freely? Was there a dominant person, or was it more democratic, with everyone sharing their ideas?

**Lily:** Quite democratic. Yes, quite democratic. Basically, everyone annotated wherever they wanted, thought of whatever they wanted. In the end, we compiled everyone's annotations, saw which points we hadn't thought of or which different perspectives we could combine for the class presentation. Or if there were any mistakes, group members would point them out. For example, disagreements over certain words or concepts could be discussed, which I think is very good.

**Alice:** Can you give an example? Like a specific point where people had differing opinions and discussed it? Do you remember?

**Lily:** I vaguely remember. Our group did Associative Annotation. It was about that "Job Hunting" article, methods for finding a job. One group member listed some methods using apps, but the apps he listed weren't very mainstream, I felt. So I suggested he change them because the more mainstream apps now are like Boss Zhipin and such. The ones he listed weren't very well-known, so I wanted him to swap them.

**Alice:** And in the end, did he listen to you?

**Lily:** Yes, he did. Quite amicably.

**Alice:** And maybe through discussion, it's easier to accept others' viewpoints.

**Lily:** Exactly, exactly.

**Alice:** I wanted to ask, sorry I said ten minutes, but I think it might go over. We're already past time.

**Lily:** No problem, no problem, no problem. It's fine, it's fine.

**Alice:** Because I really want to know what everyone thinks. So let's take it slow, okay? We'll stretch the time a bit. Do you feel that before this annotated reading, your reading methods – including pre-reading and post-reading strategies – have changed at all? Specifically for reading English articles. How did you read before, and how do you read now? Take the textbook for example.

**Lily:** Before, with the textbook, I probably wouldn't read it much at all, really. Last academic year... I really found the texts in the textbook very boring. Lots of unfamiliar words. I'd try to read a bit, find it very difficult, and then not want to continue.

**Alice:** You basically didn't read much. What about now?

**Lily:** Yes. Now the progress is that I force myself to read. I have to think about it, read it twice and think. But I think the most important point might be that my way of thinking is based on my annotation task to guide my thoughts. For example, with Associative Annotation, I pay more attention to the background of the text and the ideas the author expresses, then I expand from there.

**Alice:** So whichever point the teacher assigned, you paid special attention to that point, right?

**Lily:** Pay special attention to that point. Yes. If it were another group's type, like Writing Technique Annotation, they might focus more on writing techniques.

**Alice:** Exactly, it's task-oriented. Do you feel that your group did it once, presented only once in class, right?

**Lily:** Yes. Exactly.

**Alice:** And then, when you read other articles later, would you also pay attention to that point? Can you predict?

**Lily:** Maybe. Yeah.

**Alice:** This question might be hard to answer. I might need to ask students who did two presentations. The first time they did one type of annotation. The next time, they weren't asked to, but when reading the next article, would they still focus on that type of annotation?

**Lily:** I feel, personally, probably not. Because, well... We college students are like that, task-oriented. You tell me to do something, I do it. If you don't tell me to, I won't. Especially for English.

**Alice:** Because it's not a core subject, right?

**Lily:** Yes. Also, on the one hand, the texts... I really feel... Well, I think they're a bit detached from reality. Or, they're boring. And lots of new words.

**Alice:** The entire set of textbooks, including from your freshman year, is this Voyages series? Do you always get that feeling? It's like some professors think being academic or classic is good for you, but they ignore the fun factor. I think there should be more narrative ones, or ones that people are interested in, right? Things college students would be interested in. The article selection really wasn't great. I was forced into it too, so I had to create a lot of... wasn't it... printing worksheets for everyone in advance, having you answer the questions first, right? Otherwise, it's hard to move the class forward. If I just lecture, no one is interested, and you don't learn anything yourselves.

**Lily:** Exactly, exactly. Yes, yes. So I think you are a very responsible teacher. Compared to last semester... Not that that teacher was irresponsible... How should I put it... I can't quite explain, but he was responsible just for lecturing. He didn't think about how to improve our autonomy.

**Alice:** Okay, thanks for the recognition <laugh>. So, this question: Do you think annotated reading helps with reading comprehension?

**Lily:** Definitely. Anyway, being task-oriented, it definitely makes you understand the article more deeply. I need to know what it talks about first, then I need to infer. Since I have to annotate, I also need to think about the underlying ideas... what the intention is, or what the author wanted to express.

**Alice:** It makes you think more deeply, right? No longer limited to just the story. If it's a narrative, you don't just think, "Oh, this is the story," you might think about other aspects, like the author's background.

**Lily:** Yes. Exactly, exactly, exactly.

**Alice:** Or the structure of their ideas.

**Alice:** That's what I think <laughs>. I don't know if you feel the same.

**Lily:** Yes, yes, yes.

**Alice:** It helps you gain a more comprehensive understanding of the text, right?

**Lily:** Yes.

**Alice:** I've read some articles that say this annotated reading method can also improve students' reading test scores. So, I wanted to give an example. Do you think, since we did those three CET-4 reading exercises this semester... Do you think it helped your reading?

**Lily:** I'm not very clear if the help came from annotated reading in class or from... because I'm preparing for CET-6 this semester.

**Alice:** Yes.

**Lily:** We did those exercises two times. My feeling across those times is that it got easier and I got faster. Personally, I'd attribute it more to the vocabulary growth and improved language sense from practicing for CET-6.

**Lily:** Of course, annotated reading definitely helps because you read more and read the text more deeply, so your language sense and even vocabulary definitely improve.

**Alice:** Yes, but it might not be the only reason, right? Yes, I think so too. That's why when I read some articles claiming it improves scores, like post-test scores are better than pre-test because of it, I question whether it has that big an effect.

**Lily:** Anyway, any article you read, if you read it carefully, it will have an effect. Because if you read carefully, it definitely helps vocabulary and language sense.

**Alice:** For instance, when doing CET-4 reading exercises – taking those as an example – did you feel any difference in your approach between the last time and the first time at the beginning of the semester?

**Lily:** Personally, I don't think so. I might be set in my ways. Because I think Chinese students grow up doing exercises. From high school to university, my approach has basically continued the same, for English.

**Lily:** That is... first, to be blunt, for exams, you read the questions first, then find the corresponding content based on the questions, and match the options. That's how it's always done.

**Alice:** Yes, or analyze and discriminate <laughs>.

**Lily:** Exactly, exactly, exactly! Because, well... it's just for exams. Since the teacher limits the time, we can't afford to pay too much attention to the article's content. Basically, a day after finishing the questions, I've forgotten what the article was about.

**Lily:** Yes, we only care if the answers are correct. We don't have time to think about other, deeper content.

**Alice:** This question might be a bit repetitive... The interview design team had some overlap. But consider this: Do you feel it has changed your thinking or cognition? Through annotated reading, any change in your thinking or cognition? "Cognition" is broad. For example, your thinking process – does it make you look at an article more comprehensively? Think about it more?

**Lily:** It does. But the reason I say "might" is that I think we've only done it for one semester, so the effect might not be very significant. And you know how English class is, only two periods per week. And often, some of that time is used for exercises. So I think maybe long-term there could be an effect, but short-term, I haven't felt anything particularly significant yet.

**Alice:** Okay. Regarding this experiment with annotated reading, what difficulties or challenges did you encounter? What did you find hard or uncomfortable?

**Lily:** Personally, I don't think so... probably not? Because once you divide the work, it's not too heavy a task. For college students, I think... well, how to put it, teacher... We college students are really lazy. But I think this annotated reading is genuinely good because it improves our learning autonomy. Without it... I feel we really wouldn't learn much. Just listen in class. With it... A difficulty I find is a bit of a trade-off. Because we were divided into groups, with different methods like Associative Annotation, Writing Technique Annotation, etc. With this division of labor, we specifically think about certain aspects according to our assigned task. So I think it's not comprehensive enough. But if you want students to understand the article more comprehensively, you'd have to not divide the labor. But then the workload might be too high. It's a bit of a trade-off.

**Alice:** I considered that too. I thought if I don't specify, you can annotate freely. But I worried that free annotation might lack direction.

**Lily:** Free annotation wouldn't work. No, free annotation wouldn't work. I think free annotation might be even more chaotic. Harder to think about a single point.

**Alice:** I wanted different groups to experience different points, then hear the points from other groups. Maybe the ten groups that did two rounds might have different feelings. The later groups only did one round. I know this framework isn't perfect <laughs>. Lots of problems. But I couldn't think of a better way.

**Lily:** True. I don't have one either. But I think this is the best method so far. For now, better than nothing.

**Alice:** Okay. How did you think of the software? Were there problems with it?

**Lily:** This annotation software?

**Alice:** We used Tencent Meeting... No, Tencent Document, right?

**Lily:** Personally, I think it was fine. I didn't notice any problems.

**Alice:** Okay. Because abroad – I've read some articles – contexts are different, tools are different. They might have more intelligent ones, like Weibo, where you can like, repost, do various things. But the free one we could find... was just this one <laughs>. It's free and has decent features.

**Lily:** It's fine. It's sufficient, actually.

**Alice:** Okay. One more question at the end. Throughout this annotated reading process, is there anything that left a strong impression on you?

**Lily:** Strong impression? Not really... Does that mean I wasn't very engaged?

**Alice:** <laughs> No, no. Just anything you still remember from the annotated reading process. Could be your own, your group's, or other classmates'.

**Lily:** At the beginning... how should I say... Teacher, I have to reflect on us college students' laziness again. At first, I really didn't want to read. I couldn't get through the text because I hadn't developed the habit in freshman year. I found it a bit frustrating. But later, I learned things, and seeing how diligent and responsible you were, I found it... the impressive point might be that I learned a lot myself. Going through the semester's texts, I could get a general idea, unlike freshman year where I felt like I learned nothing. Also, the cooperation method was quite good. Because each group divided the work and presented, I felt I listened more attentively and could absorb different methods and ideas. The points they highlighted in the text helped me understand the article more deeply, more comprehensively. I thought it was good. But if we'd done it like last semester without annotated reading, I probably wouldn't have delved into things like rhetorical devices. I don't know if the teacher mentioned them in class, but I probably didn't hear or forgot, no impression. But if a student presents, I pay attention to it and underline it.

**Alice:** Okay. So the learning process, from your description, initially might have been a bit resistant, a bit painful, because you had to read. But later, did it become easier?

**Lily:** Yes, yes, yes, yes. It did. Gradually got used to it, and I realized it's a really good method. Honestly.

**Alice:** It got easier later, right? You accepted it.

**Alice:** Okay, that's everything I could think of. Sorry for taking your time, but I'm quite happy. Personally happy. I know you are a very conscientious student, and it gives me comfort, makes me feel I accomplished something this semester, that I was somewhat useful.

**Lily:** The teacher's good intentions are clear. It's because you yourself... how to say... I think you are a very dedicated teacher. Not that other teachers aren't dedicated. But maybe they want to but don't have better methods, or maybe they just haven't implemented them.

**Alice:** They might not be aware.

**Lily:** Anyway, compared to other courses, basically the teacher lectures, and there's little interaction between the teacher and students. Other classes, I mean. Of course... I don't know how to say it. I think sometimes the teacher feels helpless but can't find a way. Actually, students are the same, we feel helpless too. Sometimes the texts are boring and we can't muster the initiative to learn, but there's no solution.

**Lily:** I think English class has made a big breakthrough, really.

**Alice:** Besides, when groups presented their annotated reading content in class, would you listen? Would you note down or follow up on interesting points presented by classmates either during class or after?

**Lily:** Compared to other general education courses, in English class, I pay more attention to listening, in-class exercises, answering questions, and following the teacher's requirements. If I hear interesting annotation points, I do take them down.

**Alice:** <laughs> Thanks, thanks. Okay. So, could you add me on WeChat? Then if I think of more questions later, or want to go deeper, or ask something I missed, we can connect there.

**Lily:** Okay. I can. I can use two platforms at the same time right now.

**Alice:** Okay. Then add me on WeChat.

**Lily:** Okay.

**Alice:** Thanks. See you later then, bye!

**Lily:** You're welcome. Okay, okay, bye!

**Alice:** A quick follow-up: Do you read the annotations your classmates make on the online Tencent Document?

**Lily:** I basically would read them all, though quickly, not super carefully. Most of the annotations are short and not too complex, so it's actually quite fast to go through them.

**Interview with Rose**

**Alice:** Can you hear me clearly? My upstairs neighbor is renovating, so it's a bit noisy. Okay, good, good. Let's begin. Can you go home right after your exam today?

**Rose:** I can hear you, teacher. Yes, I just finished the last final-exam.

**Alice:** Wow, great. The year is wrapping up.

**Alice:** Okay. Today, I mainly want to talk about the annotated reading we did this semester. First, before we started this semester, had you encountered annotated reading before?

**Rose:** I encountered this method before in primary and secondary school, during Chinese class, but never in English class.

**Alice:** In primary school, what content did the teacher usually ask you to annotate?

**Rose:** In primary school, mainly new words, phrases, idioms, and good sentences or phrases.

**Alice:** How about in middle school? What did the teacher ask for then?

**Rose:** In middle school, mainly focusing on sentences that needed appreciation or analysis, things like that.

**Alice:** Trying to write down thoughts?

**Rose:** Brief comments, and text appreciation.

**Alice:** So annotated reading in English is a first-time experience, right? Okay. Through this semester's annotated reading, what do you feel you have earned?

**Rose:** I think first, this annotated reading helps me get into the context of the article relatively quickly. Then, for non-English majors like us, it can have a good effect on English reading ability. Also, annotated reading is divided into different types, like imitation, appreciation, etc. This kind of annotation helps us quickly focus on things worth learning. Without annotated reading, we might easily overlook them.

**Alice:** When you do individual annotations, how did you do it? Since we had an assignment where everyone had to annotate individually and take a photo to show me. What steps do you usually follow?

**Rose:** Because it's English, I prefer to read through the text first to get a general understanding of its meaning, then divide it into sections based on paragraph structure. After that, I will read it carefully in the third round*.*

**Alice:** Detailed reading. But do you also look up vocabulary or note down points you observe while reading?

**Rose:** That happens during the first read-through.

**Alice:** You look up vocabulary in the first pass?

**Rose:** To understand the meaning.

**Alice:** Okay. How did you usually prepare for your freshman English classes before? Because this annotated reading is essentially a form of preparation, right?

**Rose:** In freshman year, I basically condensed the three passes into one: read it once, know the gist, and stop there.

**Alice:** So the idea is to get you to invest more time and think more about the article, right. I want to ask, when your group did the annotations, how many rounds of tasks were you assigned?

**Rose:** First, everyone had to understand it individually. For collaboration, we mainly communicated. For example, with Appreciative Annotation, we'd see what sentences everyone found, then divide them into different sections and points. Then we'd work together on the presentation PPT.

**Alice:** I mean, some groups were assigned presentation tasks twice, some only once. How many times was your group assigned?

**Rose:** I think twice. Twice. Yes.

**Alice:** And the tasks were different each time, right? Once it was appreciation?

**Rose:** Appreciation, imitation, and supplementary – we had all kinds.

**Alice:** Okay. Do you feel that when you were assigned a task, like imitation or supplementary, the next time you read, you would consciously think, "Oh, I could imitate this" or "I could add something here"? Did you get that feeling?

**Rose:** Yes, yes. Actually, for every type of annotation. Because when we first started annotated reading, we roughly familiarized ourselves with each type. When reading, I pay attention to all of them, but maybe not as comprehensively as when specifically assigned a task. But I do notice them.

**Alice:** You pay more attention when assigned the task, right? Okay. How did your group usually operate? Everyone did their own, then gathered to discuss the task and everyone's ideas? Did you typically select which points to present, or did you present everyone's?

**Rose:** Yes. We presented everyone's points. Because we felt if someone found something, it must have some merit. Presenting it could also help stimulate others' thinking. So we presented everything.

**Alice:** Yes, in case some classmates hadn't thought of that point, right? Okay. You presented it in class... I think the second time the teacher drew names, right? The first time was by recommendation. Who was recommended? The group leader? Or volunteer?

**Rose:** We drew lots.

**Alice:** The first time your group drew internally?

**Rose:** Yes, the first time. Also, our group didn't really have a designated team leader because all four of us were quite proactive.

**Alice:** Wasn't it you? <laughs> Did you present?

**Rose:** No, no, no. We drew lots and another classmate did it.

**Alice:** Okay. Next question: Do you feel your reading methods or strategies changed before and after annotated reading? Compared to freshman year.

**Rose:** Methodologically, I think it's similar. It's basically the same as the reading training from high school. It's just a matter of how much attention you pay.

**Alice:** You started paying attention to more aspects. More things to think about or look up, right? Okay. Do you think annotated reading helps with your reading?

**Rose:** I think it's quite helpful, especially in university. Because for non-English majors, you don't spend as much time on English as in high school. So without reading, you get rusty. Having the annotated reading task acts as an external force to help you practice your reading ability.

**Alice:** Yesterday I interviewed a student who made an interesting point. She said college students these days are very lazy. If I, the teacher, don't require it, she won't read, she won't annotate. Do you think that's the case?

**Rose:** That's definitely true.

**Alice:** So it provides some motivation or pressure, right?

**Rose:** Yes, yes. Relying on other-discipline (opposite to self-discipline).

**Alice:** Yes, yes, other-discipline. So it's helpful for reading and learning. Do you think this method helps with doing test questions? For example, when preparing for CET-4/6. Do you feel it has any subtle influence?

**Rose:** I think the effect isn't very obvious. Because test-taking aims for efficiency: speed and accuracy. But this annotated reading, I think, is more suitable for extended English learning activities.

**Alice:** For deeper or more comprehensive understanding?

**Rose:** Yes. Yes, deep and more comprehensive!

**Alice:** Right. With timed tests wanting correct answers, you can't think too much. Okay. Do you think this method helps with your cognition or brings any changes? "Cognition" is broad. Try to answer first, and if you do not understand, I'll give examples.

**Rose:** In terms of cognition, yes. Mainly with types like Supplementary Annotation. It requires you to expand your thinking, so you might learn more or actively think about the issue.

**Alice:** Actively thinking about the issue means improving learning initiative, right? Did you encounter any difficulties or challenges during the annotated reading process?

**Rose:** I think a lot of it was not understanding the context or culture. The gap compared to Chinese.

**Alice:** So language aspects? Not knowing how to annotate? Or...?

**Rose:** Yes. Yes. Sometimes do not understanding the meaning itself.

**Alice:** Right, the content. Language creates comprehension barriers. What about the platform? Was it okay? We used... Tencent Document.

**Rose:** I think this document platform... it's a bit inconvenient because where annotations overlap, it's troublesome to click on.

**Alice:** And what bothered me was if I wanted to @ (it allows somebody to direct a message to a specific person in a group chat) another student , I couldn't do it directly. Right, yes. I find that is not good. But the only tool we could find for now is this one. It's different abroad, they seem to have other similar ones.

**Rose:** Yes, yes, <laughs>.

**Alice:** But other aspects are okay. Let me summarize: One difficulty is cultural or content/language barriers, making comprehension hard, leading to annotation difficulties. Also, the platform isn't very convenient.

**Alice:** Two more questions. One is, throughout implementing annotated reading, is there anything particularly memorable for you?

**Rose:** Memorable... I think the whole process was quite memorable.

**Alice:** It was a long process.

**Rose:** Because basically, most of our English class time revolved around this annotated reading, plus some homework. The process involved a lot of collaboration.

**Rose:** Yes. Probably the collaboration within the group left the deepest impression.

**Alice:** Okay. Tell me about it. Memorable things from your group collaboration <laughs> or scenarios?

**Rose:** For example, at the very beginning when we started annotating, everyone was very inexperienced. It was hard to find points, and we got anxious, wondering what to do. We discussed the situation. Eventually, after reading the text a few more times, we included points that seemed somewhat relevant.

**Alice:** So internal group collaboration left an impression, overcoming problems together. What about the online part? Where each group posted their annotations, and you could also write or reply to others. Did you find that process helpful?

**Rose:** Actually, I think apart from the members of the groups assigned tasks, who were quite active in writing, other students weren't as proactive.

**Alice:** Yes, yes. That's a concern I had too. The motivation wasn't strong. Maybe it's related to... how to say...

**Alice:** I saw that many students wrote a lot in the homework they photographed for me. But maybe they were shy or lacked motivation to put too much content on the shared doc.

**Alice:** Next question: Did you read what other students wrote? I think I set aside some class time for everyone to read them. Did it help broaden your thinking?

**Rose:** I found it quite helpful. Other students really thought of points I wouldn't. Some students were very meticulous, writing up a lot of details, materials, or their thoughts. I found it very helpful.

**Alice:** Okay, good. Last question: What emotional experience did this annotated reading process bring you? Did you find it painful? Or okay? Or something else?

**Rose:** At the beginning of this work, I found it quite painful, because it was unfamiliar. I hadn't put much effort into reading in freshman year, so I was already unfamiliar with the English language itself. Adding the unfamiliarity of annotated reading made it difficult and time-consuming. But later, because I was preparing for CET-4 this semester, along with reading the texts, my language sense improved, and it felt easier. I also became more familiar with annotated reading. Like I said earlier, I paid more attention. It took less time and felt easier. Towards the end, that was the case.

**Alice:** You didn't know how to annotate, pressure, pain. Later, through group work, class presentations, teacher explanations, it got easier. Is that right?

**Rose:** Yes. Yes, that's right.

**Alice:** You mentioned a point earlier. Because annotated reading requires several reads, it subtly helps with language sense. So for CET-4/6 reading, there might still be a slight subtle influence?

**Rose:** I think there is indeed an effect. Without it, I'd probably do very little (English) reading on my own. Again, it's “other-discipline”.

**Alice:** Okay. That's all the questions I have for now. Could you add me on WeChat? If I think of more questions later...

**Rose:** Okay.

**Alice:** ...to follow up, I can message you directly on WeChat. It might be faster for you to see the message.

**Rose:** Okay, okay.

**Alice:** Great. Thanks, Rose. Thank you very much! Okay, have a great winter break!

**Rose:** Okay, thank you teacher! Thank you teacher!

Follow-up Question: Do you read other students' annotations on the online Tencent Document carefully?

If my group was assigned a specific task, I read every comment carefully. Otherwise, I’d just scan to see if others’ findings aligned with mine.

Alice: When you discuss and summarize group annotations, do you communicate face-to-face offline or online through apps like WeChat?

Rose: Students in the same dormitory will communicate face-to-face offline, but if there are students from other dorms in the group, we'll do it online. Our group directly communicates in the WeChat group created by our roommates. It really depends on which method is more convenient.

**Interview with Emily**

**Alice:** Okay, let's get straight to the point so I don't hinder your other tasks. First, thank you for participating in this interview. It gives me a chance to understand your views on the annotated reading framework we implemented this semester and know what you're thinking. Before this semester started, had you encountered annotated reading?

**Emily:** Probably the most similar thing was maybe like in primary or middle school, when preparing a text, the teacher might ask you to annotate or mark things, but not like now where we separate them into specific types like Associative Annotation. Not with so many categories, nor thinking about a single category for annotation.

**Alice:** So in Chinese, the language arts subject, quite a while ago, in primary and middle school, you tried some basic methods, right? But English annotation is only being introduced this semester. Is that right?

**Emily:** More or less.

**Alice:** Through annotated reading, what do you feel you learned?

**Emily:** For example, I was assigned Supplementary Annotation. So I would focus specifically on the supplementary category when thinking about the text – its background, a word, where the author's viewpoint came from. It leads to a more comprehensive analysis of the text.

**Alice:** Not just more comprehensive, but deeper, right?

**Emily:** You need to find information, so you learn more.

**Alice:** Okay. Anything else besides that?

**Emily:** I haven't thought of it yet.

**Alice:** Let's move on to the next question. We can come back later. In the annotated reading process, there were two assignments. First, individuals annotate their own books and upload photos to the homework platform. Second, groups were assigned a topic to work on together. How did you do your personal annotations? How did you complete the requirement to upload a photo? What steps did you take?

**Emily:** Generally, I usually read through it first. Then, on the second reading, I will deliberately look for parts that our team was assigned to annotate. I will also search online (for relevant information) at the same time.

**Alice:** Did you also look up vocabulary during the first pass?

**Emily:** Yes, I check words while reading the text.

**Alice:** First pass deals with difficult words or sentences. Second pass focuses on the assigned task, searching and thinking. Is that it?

**Emily:** Yes.

**Alice:** Basically two passes, right?

**Emily:** If I can't find enough points, I would read it again for a final check.

**Alice:** If difficult, read it again. How did you do the group annotations?

**Emily:** We usually...after paper-based annotation, we would report our findings for collective review. When information was abundant, we discussed which parts were the most valuable and refined the content to make it more concise.

**Alice:** So you needed to select and refine. You discussed democratically which ones were suitable for presenting. Did you have a group leader, or was it democratic discussion?

**Emily:** No, we just... we just looked at them.

**Alice:** So everyone was quite organized. Even without a leader, you collaborated to complete the task.

**Emily:** Yes.

**Alice:** After this annotated reading, have your reading methods or strategies changed compared to before?

**Emily:** Definitely, I read more carefully. And because I have to annotate, I naturally think more comprehensively.

**Alice:** You think more comprehensively.

**Emily:** Before, it was more about simply understanding the content.

**Alice:** Okay. Do you think annotated reading helps with your reading?

**Emily:** Yes.

**Alice:** Can you elaborate?

**Emily:** For example, first, obviously, I get a deeper understanding of the text. Besides that, because it's group-based, when you communicate and discuss with your group members, you naturally want to hear their ideas and see how they approached annotating the text. Sometimes I can't find many points, and they seem able to analyze more comprehensively and find more.

**Alice:** It enriches your thinking, right?

**Emily:** Yes.

**Alice:** We also used Tencent Document online, where I required everyone to share at least one annotation. Did you read the content there?

**Emily:** Sometimes yes, sometimes no.

**Alice:** When you read, did it help deepen or broaden your understanding of the text?

**Emily:** Yes, because there were definitely things I hadn't thought of myself, and other students could think of them and annotate them.

**Alice:** Do you remember any specific example?

**Emily:** Let me think. For the more recent one about job hunting, I remember one student analyzed and annotated many methods and channels for job hunting, like specific apps that are good or something.

**Alice:** He mentioned different platforms or online channels, right? Which ones are more reliable or better, something like that?

**Emily:** Yes.

**Alice:** That left an impression, right? Everyone was preparing for CET-4 or CET-6 this semester. Did you take one?

**Emily:** Yes, I did.

**Alice:** CET-6 or CET-4?

**Emily:** CET-6.

**Alice:** Do you think this annotated reading helped, even a bit, with preparing for the reading section or other parts of CET-4/6?

**Emily:** I think it's just average, just okay. Not very obvious.

**Alice:** No obvious help. That's fine. This is open; the exams are over, and this won't affect your grade. I just want to know what you think.

**Emily:** I feel my reading habits are probably similar to before.

**Alice:** Facing an article you can calmly read, I feel this annotated reading might be helpful. But for test-taking, with time constraints and wanting to pick answers quickly, you probably don't think as much.

**Emily:** Yes. Usually, read the questions, options, then the text, and choose directly.

**Alice:** Okay. Do you think this method changes your thinking or cognition? Your problem-solving approach? Or something you thought was one way but turned out another?

**Emily:** When thinking, it's more targeted. Before, my scope was broader, or I wouldn't think much, just look at surface things, wouldn't think this much.

**Alice:** Before, you might just understand the content. Now you know there are different angles to view the text. Especially when your group was assigned a task, that task subtly influenced your subsequent reading, right?

**Emily:** Yes, definitely.

**Alice:** Okay. So, looking at issues more comprehensively and deeply, or when reading articles.

**Emily:** Yes.

**Alice:** During our course process – not the whole course, but the annotated reading part – did you encounter any difficulties or challenges?

**Emily:** Let me think. I think I was assigned... either Evaluation or Appreciative Annotation? I might have confused the task assigned to my group with another type, wondering if they weren't basically the same. What aspects should I focus on for this annotation? How should I analyze it?

**Alice:** The task wasn't clear enough for you, causing confusion, right?

**Emily:** Yes. I wasn't very clear on the analysis boundaries between them. Also, I felt my thinking was still limited. I probably wasn't used to or adapted to this method, so the annotations I could think of were relatively few.

**Alice:** What's that related to? Vocabulary? Knowledge base? Thinking style? Which factor do you think is most relevant?

**Emily:** Probably knowledge base. Expanding things is more difficult for me.

**Alice:** Not very familiar? How did you find the Tencent Document platform? Was it okay? Or inconvenient?

**Emily:** It was quite good. You could annotate and see others' annotations. And you could quote.

**Alice:** Yes, yes, <laughs>. You could insert links, right? Okay. Throughout this annotated reading process, is there anything particularly memorable for you?

**Emily:** Presenting in class.

**Alice:** You presented, right?

**Emily:** I happened to be drawn.

**Alice:** So your group only did it once, right? Only one assigned task, or two?

**Emily:** We had... initially there was one time we needed to make a PPT. Then later with Tencent Document, we were drawn only once.

**Alice:** And you were drawn? Why is it memorable? Nervousness?

**Emily:** Yes. Because our content was quite extensive. And although we filtered for more refined content, we might have just talked about which points were good, without summarizing or refining them further. So when I presented, I had to summarize on the spot using my own words, which made me a bit nervous.

**Alice:** Nervous and excited <laughs>, wanting to do well, right?

**Emily:** Yes.

**Alice:** Okay. Overall, did you find the annotated reading process painful? Relaxed? Neutral? Or pleasant?

**Emily:** [Pretty neutral. I just treated it like a regular homework assignment, just something I had to do to prepare and read the text.

**Alice:** So for you, it was just a task, find a way to complete it?

**Emily:** Yes.

**Alice:** The reason why I ask this question is that some previously-interviewed students mentioned finding it painful initially, maybe related to text difficulty, dry topics, plus needing to read the article which was already painful, then complete the assigned task. They found it painful. For you, it was okay?

**Emily:** It was similar to previous preparation. Sometimes I found fewer points to analyze.

**Alice:** I think it might be related to your English level. Since you're preparing for CET-6, your foundation is likely good. I'm guessing <laughs>. Not asking for agreement. Okay, those are my questions for now. Can you add me on WeChat? If I think of more later, I can message you directly – might be faster. Probably everyone uses WeChat more during break than QQ. Okay, let me say...

**Emily:** Okay, thank you teacher!

**Alice:** Then you can go and enjoy yourself.

A quick follow-up: Do you read what other students annotate on Tencent Document?

**Emily:** Most of the time, yes.

**Alice:** Okay, bye!

**Alice:** When you read, do you read from beginning to end, or selectively? Are there specific parts you pay attention to?

**Emily:** I read from beginning to end. I paid special attention to comments in the sections I had annotated, curious to see what others had to say.

**Alice:** Sorry to interrupt, one more follow-up question. When your group discussed the assigned annotation type, what method did you usually use? Offline face-to-face, or via a WeChat group? Also, how was the atmosphere during discussion?

**Emily:** We mainly discussed offline face-to-face! Because all group members are roommates, direct discussion is more convenient. But sometimes we'd share ideas and found materials in the WeChat group. The atmosphere was quite good, I think. Everyone spoke their mind directly. For points that were superficial or disagreed with, we'd directly add criticism or objections.

**Alice:** Okay, thanks.

Alice: When students in our class give group presentations on annotated reading content, do you listen? During class or after class, do you take note of the interesting content your classmates share or further explore it?

Emily:When classmates share annotations in class, I listen to them, but I don't take notes or explore further.

**Interview with George**

**Alice:** Did you receive the notification that recording has started?

**George:** Yes.

**Alice:** Good, good. That means it's working. Let's chat casually first. Yesterday you seemed nervous. First interview?

**George:** Yes.

**Alice:** Interviews are simple. The interviewer asks, you answer. No special preparation needed, I think, unless specified. I've interviewed five students so far and got very useful information. You're my last interviewee, so please speak openly. No need to worry about critiquing the class or the annotated reading – point out any flaws. Exams are over, and I will grade fairly. This interview helps me, so I'm grateful. Don't hesitate to mention bad points.

**Alice:** Let's begin. This semester, besides regular textbook content, we did an activity: annotated reading.

**George:** Okay.

**Alice:** Before this semester, had you encountered annotated reading?

**George:** Possibly before in Chinese class, during middle or high school, annotated reading for Chinese texts.

**Alice:** What aspects did the teacher usually ask you to annotate?

**George:** Anything. Anything you wanted. Your own thoughts, etc. It’s very broad. They'd just check how much you annotated in the end.

**Alice:** Just required you to annotate, no specific focus, right?

**George:** Yes, low requirements.

**Alice:** So this semester is your first time with English annotated reading, with more requirements, right?

**George:** Quite novel.

**Alice:** What do you feel you learned from annotated reading?

**George:** An obvious point is I became more proficient with some online platforms and tools. Because my English might not be very good, I needed to use online learning platforms to assist my annotations.

**Alice:** For example?

**George:** For example... what's it called...Youdao Dictionary.

**Alice:** Lots of vocabulary, difficulty, some unknown words, so you use Youdao Dictionary (an online dictionary that supports Chinese-English and English-Chinese lookup) to look them up.

**George:** Yes. Also, sometimes Baidu (a search engine) for other things.

**Alice:** Yes, Youdao for words, Baidu or other search engines for extra information, right?

**George:** Also, compared to previous methods, I think it improved my autonomous learning ability a bit.

**Alice:** How so?

**George:** Including the increased proficiency with learning software mentioned earlier, that's a benefit of self-learning. I think it also gives me more sense of participation in English learning. For example, analyzing textbook vocabulary and sentences myself, overcoming small difficulties, gives me a sense of achievement. Also, compared to before, my memory is deeper.

**Alice:** You mentioned "small difficulties." What kind of small difficulties?

**George:** Sentences. Some sentences, you might know every word but still can't accurately express the meaning.

**Alice:** Can't understand exactly what it means, right?

**George:** Yes. Then I need to consult and search.

**Alice:** Consult classmates or just use online platforms, electronic methods?

**George:** I usually use electronic methods.

**Alice:** Okay. Regarding the homework assignments for annotated reading: one was individual annotation, requiring a photo upload to the mini-program. The other was group task. How did you do the individual annotation part?

**George:** For individual annotation, our group mainly did Evaluative Annotation, Appreciative Annotation, and Questioning Annotation.

**Alice:** First, the teacher assigned an assignment – for example, for Unit 1 or 2 – requiring individual annotations and uploading a photo before class as an assignment. What steps did you take to complete it?

**George:** I would read through the whole text first to understand its meaning, then do some annotations based on the specific content of the assigned task.

**Alice:** Still guided by the teacher's assigned task, right?

**George:** Yes. Understand it first, then do (the annotated reading task).

**Alice:** Read through the text once, get to know the main content, then focus on the task assigned to your group to find relevant parts, right? Then annotate and upload. Is that the process?

**George:** Then we have group discussions.

**Alice:** Yes. Next is the group stage. Did you have a leader, or was it democratic and spontaneous to complete the task?

**George:** The leader's presence felt quite low.

**Alice:** So there was a leader, but...

**George:** But he seemed more like a member.

**Alice:** <laughs> So who organized it? Someone had to find time for everyone to do it.

**George:** Yes, one student would proactively take charge, and we'd meet face-to-face for discussion.

**Alice:** Someone proactive and responsible. Then for the assigned task, how did you proceed? Discussion? Other methods?

**George:** First, gather everyone's annotations and points. Then check for duplicates; if overlapping, see if they can be merged. Also, some annotations we felt were too superficial or perfunctory, so we'd delete them.

**Alice:** Too perfunctory, not comprehensive enough, vague?

**George:** Yes. Yes. Some might be a bit childish or perfunctory, so we'd delete them.

**Alice:** Your group did two rounds – first unit and maybe last unit? First unit required making a PPT and presenting in class. How was the presenter chosen?

**George:** Yes. The group leader volunteered.

**Alice:** The leader volunteered. The leader's role manifested then. The last time, the teacher drew randomly. For that random draw, everyone prepared because they knew it could be anyone?

**George:** Yes.

**George:** Everyone was quite nervous.

**Alice:** <laughs> So during group discussions, you compiled points. For the second round, did you write the discussed points in your books? On paper? Or just listen? Did you annotate them on the Tencent Doc? How did you handle the results of discussion?

**George:** Before using the online doc, we'd write them in our books. After we had the online doc, we'd write them directly on the doc.

**Alice:** One student would write them up, everyone knows that's the point, okay. After annotated reading, have your reading methods or strategies changed compared to before? Using freshman year as baseline. Without this method, how did you read texts before versus now with required annotation? Any changes in methods or strategies?

**George:** I think I have more of my own thoughts when reading. I might view the author's viewpoints more objectively. Because I need to annotate, I don't just follow the text blindly. I think about whether the author's point is good, add my own thoughts. Or whether a word choice could be better, if another word might work.

**George:** Also, whether a certain part of the text is designed reasonably. For argumentative texts, does it have supporting material? Is the story plot reasonable? Does it feel abridged, a bit strange? Thoughts like that.

**Alice:** Freshman year, the purpose of annotated reading was for deep reading, good preparation. How did you usually prepare texts in freshman year?

**George:** Freshman year, I don't think I had a habit of preparing.

**Alice:** The teacher didn't require it?

**George:** Yes. We just showed up to class.

**Alice:** <laughs> Just brought the book.

**George:** Yes. The teacher prepared everything on slides.

**Alice:** Everything ready on slides.

**George:** Yes. Relevant knowledge was ready. We just had to write it down.

**Alice:** More passive reception, right? Now with annotated reading, you think more, as mentioned – about reasonableness, main content. Your group did three types – Evaluation, Appreciation, Questioning. Later when reading, do you think more objectively or question things? Did these three types of annotation training influence you later? A conscious focus?

**George:** Yes, conscious focus. Not completely led by the text. Adding my own thoughts.

**Alice:** I mentioned in class that I feel this way. I wonder if students do too. For example, doing Questioning Annotation, I consciously note whether something makes sense, if there's doubt, I write it down. Do you share that feeling?

**George:** Yes, yes.

**Alice:** Do you think annotated reading helps with our reading? Using the textbook as an example.

**George:** Using the textbook, it greatly helps me not to get bogged down in individual words.

**Alice:** What do you mean? Explain a little bit.

**George:** Before, I might struggle over a single word, trying to translate it but not well, always obsessing over details.

**Alice:** Is this related to vocabulary? The vocabulary is not enough, so you want to understand each word?

**George:** Yes, vocabulary is one aspect. Also, even knowing the meaning, after translation, it doesn’t feel right.

**Alice:** Not considering context?

**George:** Yes. No English context. Now I put more energy into more meaningful things. Obsessing over words costs me time, and in the end, I don't feel I gained much, just understood that one sentence.

**Alice:** What more meaningful things do you mean?

**George:** For example, the aesthetic beauty of the language, the quality of its structure, the author's emotional expression.

**Alice:** More macro, viewing from an overall perspective. Another question, perhaps not directly related to annotated reading: Did you prepare for CET-4/6 this semester?

**George:** Yes, I did.

**Alice:** CET-4 or CET-6?

**George:** CET-6.

**Alice:** Wow, that implies a decent level <laughs>. Passed CET-4 at the first try? Do you think annotated reading helps with doing CET-4/6 exam questions?

**George:** Actually, I separate daily learning from exam preparation. Daily study is daily study. Exams are exams. Strategies differ.

**Alice:** For CET-6 reading, what's your strategy? How do you prepare?

**George:** Similar to CET-4. Memorize vocabulary first, then do practice questions.

**Alice:** Memorize CET-6 vocabulary, then do practice tests, real exams? Is that it?

**George:** Yes. Yes. Do a bit of each section daily.

**Alice:** Okay. Let me think whether there are any other questions. Annotated reading has various types. Do you think it impacts your thinking or cognition?

**George:** Probably when reading an English article, I will consider more aspects, more dimensions of thought.

**Alice:** Partly because you completed tasks from different perspectives, causing subtle influence – "I should pay attention to this aspect"?

**George:** Yes. Different annotation methods provide inspiration for thinking while reading.

**Alice:** Guidance, right? "I can think from this angle." Did you encounter difficulties or challenges with annotated reading? Including platform use (Tencent Document) and class presentations. Any hard aspects?

**George:** Probably my own insufficient English level.[So it consumed quite a bit of time.

**Alice:** Time-consuming.

**George:** Yes. Plus, after-class communication with others. Some people procrastinate, some lack tact and argue. Sometimes we argued a bit and had disputes.

**Alice:** Teamwork is a difficulty. Plus your own level, making reading hard. Difficult vocabulary, uninteresting topics.

**George:** My own level is somewhat lacking.

**Alice:** Okay. The Tencent Document platform for annotating and reading others' annotations – you participated, right?

**George:** Yes.

**Alice:** How was the platform? Any issues?

**George:** The platform works a bit slowly. It felt laggy opening it. Maybe that’s because my internet speed.

**Alice:** Did you read other students' annotations?

**George:** Too many.

**Alice:** Read many? No need to recall specific content. But you read them?

**George:** I probably just listened in class during presentations. Manually clicking through was too laggy.

**Alice:** Hardware difficulty. So listening to classmates present is better? But you still completed the required one annotation yourself?

**George:** Yes. Everyone annotated.

**Alice:** Good. Throughout the annotated reading process, is there anything particularly memorable?

**George:** Anything... Probably the feeling of participating in the text myself.

**Alice:** You became part of it?

**George:** The sense of participation and achievement stands out as most memorable.

**Alice:** You became the master, exploring, thinking, evaluating?

**George:** Yes. That feeling is really good.

**Alice:** I'm glad <laughs> you felt a sense of achievement. So personal achievement is memorable. We did annotated reading based on the Text As of four texts. Was it painful initially? Indifferent? Pleasant? Did feelings change over time?

**George:** Initially, I didn't know where to start. No prior exposure. My first type was Evaluative Annotation. I didn't know how to evaluate. Do I just say it's good or bad?

**Alice:** Didn't know the standard. Then?

**George:** We wondered if the teacher could provide a template, examples from other texts, so we'd know.

**Alice:** Good suggestion. But I think not giving one is fine. If you think it's good, say so. Your personal view.

**George:** Yes, that works too.

**Alice:** Painful and confused initially. Later, did it improve?

**George:** Yes. Later, through discussions, I gradually learned the general direction. Maybe not exact, but I had my own ideas.

**Alice:** Okay. So later it became easier?

**George:** Yes, more proficient.

**Alice:** Okay. I've asked my prepared questions. I got some perspectives different from other students – interesting and good. Thank you, George. I have your WeChat; if I have follow-ups, I'll message you.

**George:** Okay.

**Alice:** Roughly how much time did you typically spend on individual annotations for each unit?

**George:** About 20 minutes for the initial read-through. Then roughly 1 hour to look up information for specific words and sentences. Then about 30-45 minutes to complete the individual annotations based on the assigned task. It fluctuates a bit depending on text length and task requirements.

**Alice:** When groups presented annotated reading content in class, would you listen? Would you note down or follow up on interesting points?

**George:** I do listen during group presentations. Especially for parts I annotated carefully myself, I listen more attentively. But sometimes if a group hasn't prepared well, I listen less attentively. Interesting or new points I note down. Also, because I'm a bit of a perfectionist and detail-oriented, if I have an unresolved question, it bothers me. But sometimes I end up spending too much time on it, affecting efficiency.

**Interview with Jack**

**Alice:** To save time and not affect your fun time later, let's start.

**Jack:** Okay, okay.

**Alice:** Sorry to interrupt. This semester we experimented with annotated reading. Before this semester, had you encountered annotated reading?

**Jack:** No problem, it's fine. No.

**Alice:** Not even in English? Or never? Some students mentioned in primary/middle school, Chinese teachers asked to mark key words/sentences or write thoughts. You've done some practice?

**Jack:** I have, but relatively little.

**Alice:** Not your strong suit? Okay. What did you learn from this semester's annotated reading?

**Jack:** I think the gain is being able to quickly summarize what each paragraph says, and also, looking at the whole text, outline its structure – basically, quickly understanding what the text is about.

**Alice:** Getting the main idea. We had two assignments: individual annotations (photos submitted) and group tasks. How did you do individual annotations?

**Jack:** For individual annotations, first, I read through the text to see what it's about, then go paragraph by paragraph, sentence by sentence to summarize.

**Alice:** Summarize the main idea. Did it usually take one read-through to annotate, or did you need to read several times <laughs>?

**Jack:** I could do it in one go. The texts seem relatively simple.

**Alice:** One go. Okay. Anything else to say?

**Jack:** No, that's it.

**Alice:** During group annotations, how many times was your group assigned a task?

**Jack:** Twice, I think.

**Alice:** First unit and last unit?

**Jack:** I don't remember the count exactly. I remember two times.

**Alice:** Okay. When your group worked on the assigned task, how did you do it? How did you produce the group assignment?

**Jack:** We would divide the work based on the task. After dividing, we'd discuss offline, since we're all guys.

**Alice:** Did you have a group leader?

**Jack:** No leader.

**Alice:** Fair, friendly discussion? <laughs> How did you divide work for, say, Unit 1?

**Jack:** We'd first ask what everyone wanted to do or was good at. Usually, we'd divide it that way.

**Alice:** Everyone volunteers for strengths, then combine. For example, content, PPT, presentation?

**Jack:** Yes. Something like that.

**Alice:** Wouldn't the person doing content be under high pressure?

**Jack:** Not really. For annotated reading, we'd divide by paragraph of the text. For example, you do the first few paragraphs, I do the rest. Then, each person would create a simple slide for their part. Finally, they'd be compiled by the person who had fewer paragraphs. That person would also present.

**Alice:** So that person compiles the PPT and presents.

**Jack:** Yes. But the PPT is almost done by then, just compiling.

**Alice:** And they had no objection? Since they... haha.

**Jack:** No objection. Because that was me.

**Alice:** You presented?

**Jack:** Yes. I like presenting.

**Alice:** Okay. Thank you for presenting. Through annotated reading, did your reading methods or strategies change? Compare freshman year/high school to this semester.

**Jack:** Probably there was little change. I've developed habits. My previous methods might not change because I'm used to them.

**Alice:** You read similarly before?

**Jack:** Yes. I used my own methods before. After many attempts, I found what works for me, so I don't usually change.

**Alice:** Okay. Do you think annotated reading helps with your reading and learning?

**Jack:** Yes, it helps. Like quickly grasping the main idea of each paragraph.

**Alice:** Consciously summarizing each paragraph, then the whole text. That helps. Did you take CET-4/6 this semester?

**Jack:** Yes. I took CET-6 this semester.

**Alice:** Do you think this annotated reading helps with preparing for CET-4/6, especially reading?

**Jack:** For my CET-6 practice, annotated reading probably just helps me get the main idea of paragraphs faster. But for vocabulary, reading techniques, etc., maybe no significant improvement.

**Alice:** That's fine. Open answer. Go ahead <laughs>.

**Jack:** Also, listening practice this semester seemed a bit lacking.

**Alice:** Our class didn't do much listening practice?

**Jack:** Yes, maybe bi-weekly or tri-weekly? Too low frequency.

**Alice:** Yes.

**Jack:** Yes. From classmates, feedback is: insufficient listening time, needing self-practice. Also for writing: maybe more in-class expansion on sentence patterns, vocabulary, hot topics. Expansion seems a bit limited.

**Jack:** Maybe confined to the textbook. Expansion is a bit little. Much depends on self-practice.

**Alice:** Okay. I'll take note <laughs> and try to do better next time. This question might overlap, but it's in my outline: Did annotated reading change your thinking or cognition?

**Jack:** Yes. Getting the main idea faster.

**Alice:** Okay. During the process, did you encounter difficulties or challenges?

**Jack:** No. Grasping the main idea of each paragraph is usually intuitive. Summarizing it myself is fine.

**Jack:** I don't think it was difficult.

**Alice:** The activity wasn't difficult for you? Your English level is quite good?

**Alice:** Vocabulary and sentence structure aren't issues for you?

**Jack:** Yes. I'm quite sensitive to the meaning of each sentence.

**Alice:** So your fundamental reading comprehension ability is naturally good. Did you use the Tencent Document platform?

**Jack:** Yes, I annotated. I wrote simple summaries in Chinese or English for each paragraph.

**Alice:** Summaries? I noticed you didn't do the final reflection assignment that everyone did (different from this interview). Do you remember your username on Tencent Document?

**Jack:** Not so much. Which assignment was that?

**Alice:** Could you check and tell me later? No problem. Just log in and look at past Document. Your nickname appears when you write. Everyone used nicknames.

**Jack:** Okay. I'll find it later.

**Alice:** If you didn't write, that's fine. Exams are over. I'm just curious about your nickname, to see your annotations.

**Jack:** I annotated on each text's doc. My nickname was **, I think.

**Alice: ****? I remember that name. You annotated many times. No further questions then <laughs>.

**Jack:** Okay, okay.

**Alice:** Using the Tencent Document platform, did you find it inconvenient or problematic?

**Jack:** I used my phone to log in it. Maybe there is a problem with my phone--it lagged a bit. Not as good as paper, but fine overall.

**Alice:** Okay. During the annotated reading process, anything memorable?

**Jack:** Not really. My group finished all work very happily and smoothly.

**Alice:** <laughs> Very happily. So overall pleasant? Emotional experience was...

**Jack:** Relatively calm.

**Alice:** Calm. Unlike others who found it painful <laughs>. Needing multiple reads to get content, annotate, discuss.

**Jack:** I saw some classmates using phone translation apps to take photos and translate directly. Maybe matching Chinese to English text was troublesome. Finding sentences for annotation might be hard.

**Alice:** Other students did that?

**Jack:** Some students did.

**Alice:** You read English directly?

**Jack:** I usually read the text directly, in English, finding keywords in each paragraph.

**Alice:** (Echo issue) You read English first, underline keywords, summarize paragraphs. If stuck, do you check WeLearn translations or use phone?

**Jack:** Use phone directly.

**Alice:** Look up words directly. Okay. That's my questions for now. If I have follow-ups, may I add your WeChat?

**Jack:** Yes, no problem.

**Jack:** Also, some classmates feel the course lacks integration with CET-4/6 content.

**Alice:** They want more test-oriented integration?

**Jack:** Yes. As class representative, I know some classmates have passed CET-4, others preparing for CET-6. Those preparing for CET-6 feel vocabulary is lacking, not enough expansion. They self-study daily. CET-4 students feel writing and listening are insufficient. Listening frequency is low. Writing needs more hot-topic vocabulary, exam techniques, useful phrases/patterns to impress graders – more depth.

**Alice:** Okay, thanks for the suggestions. We'll stop here.

**Jack:** Okay. Thanks for your hard work, teacher!

**Alice:** No, thank you for yours. We'll talk later. You can leave the meeting.

**Jack:** No problem. Okay.

**Follow-up Question:**

**Alice:** Do you read other students' annotations on Tencent Document carefully?

**Jack:** No. On one hand, reading long sentences on my phone is dizzying. On the other hand, tasks other groups were responsible for weren't closely related to our group's task, so I didn't pay much attention.

**Interview with Kate**

**Alice:** I'll start recording now, then convert audio to text. Let's get straight to the point.

**Kate:** Okay.

**Alice:** This interview is about your views on this semester's annotated reading. First, had you encountered annotated reading before this semester?

**Kate:** For English, no. I haven't encountered annotated reading.

**Alice:** But you have in Chinese?

**Kate:** Yes, yes. Much more in Chinese.

**Alice:** When did Chinese teachers start asking for annotations or underlining?

**Kate:** Starting from second grade of primary school. That teacher was very strict, so I developed the habit of annotating in Chinese books from then on.

**Alice:** So English is first time, but you have experience in Chinese?

**Kate:** Yes.

**Alice:** We did about four units of annotated reading this semester. What do you feel you learned?

**Kate:** What did I learn? Personally...

**Alice:** Any gains?

**Kate:** <laughs> Because I had more exposure to Associative Annotation and Evaluative Annotation this semester – those were my group's assigned tasks – I was more diligent in those areas. In Unit 1, we was required to do associative annotation. I searched for relevant concepts (of species) beforehand and developed a profound comprehension of the subject. ... not just knowledge, but general facts.

**Alice:** For example?

**Kate:** I forget... Unit 1 is about the environment. For Associative Annotation, I searched for its concepts and got a deep understanding. Linking that to the text. There were species mentioned; I searched and associated (with the text), which helped expand my thinking. I found it very helpful.

**Alice:** Helpful in what way? Broader thinking? Better search skills?

**Kate:** Both. Mainly finding information.

**Alice:** You consciously searched? Before, would you have searched while reading English texts?

**Kate:** No, no habit. Because my English isn't very good. I relied heavily on translation software. It was hard to focus on reading.

**Alice:** Biggest gain is Associative Annotation?

**Kate:** Yes.

**Alice:** It left an impression, leading to conscious searching for related content. Anything else?

**Kate:** Not much else, probably.

**Alice:** You did it yourself, so it left a deeper impression?

**Kate:** Yes, yes.

**Alice:** For individual annotations (photo upload), how did you do it? Your process when you get a text?

**Kate:** When I get a text, I first read it through. I translate what I can, mark what I can't (there used to be a lot of marks). Then I check the WeLearn translation, write the Chinese, then read the whole text again. Next, as a process, I discuss with my group members to deepen impressions. Finally, I do the Associative Annotation. First, I note potential angles for annotation, then search for information, then write it in my book.

**Alice:** Usually two passes (on the individual annotated reading)?

**Kate:** Yes.

**Alice:** First pass marks difficult/unclear points. Second pass checks WeLearn for meaning, then discusses annotation points with classmates. Then writes in book?

**Kate:** Yes. Also gather some of their ideas.

**Alice:** You note down classmates' interesting points. How did group discussions go? Leader-led or free?

**Kate:** Usually free discussion. Say whatever comes to mind. If certain points seem adoptable, we use them. For Unit 1, being first and inexperienced, we divided work. We discussed together, but each person sent their point to the one making the PPT.

**Alice:** For the first presentation, groups recommended someone. How was your group's presenter chosen?

**Kate:** We drew lots. We're easygoing. Do whatever is drawn.

**Alice:** Whoever drew it presented. As an early group, did you get a second round?

**Kate:** Yes, Unit 6, Evaluative Annotation.

**Alice:** How was Evaluative Annotation?

**Kate:** Honestly, I didn't gain much from Unit 6. Reading the Unit 6 text was difficult for me. Plus, compared to Unit 1, the language in Unit 6 was cold and formal. Our group might have had limited ability; we only found 2-3 points. It is hard to extend. We wondered if the text wasn't suitable for Evaluative Annotation.

**Alice:** Possible. Text type matters. Narrative allows expanding thinking. Unrelatable, dry texts leave little to say. Okay. Comparing your freshman year reading methods to this semester, any changes in strategies or methods?

**Kate:** I'm better at finding keywords now. I actively look for the predicate, subject-verb-object. In freshman year, analyzing long sentences was hard. Now I actively grasp keywords. I can see key nouns or phrases in a paragraph.

**Alice:** More sensitive to sentence structure and keywords?

**Kate:** Yes, yes. That's quite effective for information matching questions.

**Alice:** That's the most prominent change – finding keywords better. Conscious awareness? Do you think annotated reading helps your reading and learning?

**Kate:** I think it does. Although the teacher treats it as a task/assignment, having that purposeful goal helps me immerse more. I feel I understand the text and its theme better.

**Alice:** Teacher's requirement leads to better reading. A previous student said, "We're lazy now. If you don't require it, I won't read it multiple times."

**Kate:** Yes.

**Alice:** She said in freshman year, she never prepared for the English Class and just listened (to the teacher). With this task, she reads 2-3 times or more. Do you agree?

**Kate:** I do a bit. But I'm not resentful. Because I signed up for CET-4 this year (first time). I thought this method might help my English, so I quite liked it.

**Alice:** How does it help with CET-4 preparation?

**Kate:** Reading ability improves.

**Alice:** How? More reading? More careful reading?

**Kate:** Finding keywords. Also part-of-speech recognition. Knowing some words from reading helps with writing or recognizing them elsewhere. Reading becomes smoother when encountering unfamiliar texts.

**Alice:** So detailed reading improves vocabulary and grammar, leading to better comprehension?

**Kate:** Yes.

**Alice:** So annotated reading helps with CET-4 reading?

**Kate:** Yes, yes.

**Alice:** One student today said annotated reading doesn't help test-taking. Time constraints, need for speed, picking correct answers. No perceived relationship. Another said it helps because repeated reading enhances language sense, aiding future reading. Which do you agree with?

**Kate:** The latter. I think it's useful.

**Alice:** Your reasons are vocabulary, grammar, keyword finding. Does annotated reading change your thinking or cognition?

**Kate:** As said, searching for information, asking people, learning extracurricular things.

**Alice:** Expanding knowledge. Learning from peers. Any difficulties or challenges during this process?

**Kate:** As for challenges, definitely vocabulary first. The textbook has many new words. Even with side glosses, it's hard to read while looking left/right. If not found, you look it up. Affects reading speed. Also, annotated reading (Associative Annotation) is very time-consuming. First time took 1-2 hours. Very strenuous.

**Alice:** Strenuous due to vocabulary and text difficulty? Also limited knowledge/vision on how to associate?

**Kate:** Associative Annotation, yes.

**Alice:** For the online Tencent Document activity (groups post results, others annotate at least once – despite low enthusiasm), did it help you understand texts better?

**Kate:** Bringing everyone's ideas together on the doc is like indirect discussion. You see others' thoughts. But personally, it's hard to just look at them. I might think "I agree, I agree," but to write something new based on theirs, I find it hard. Basic thoughts seem already said. Adding more feels meaningless.

**Alice:** Limited by your own [language or knowledge limitations. How did you find the Tencent Document platform?

**Kate:** A bit constrained. I rarely use my computer (QQ, a social media app, not logged in). I'm used to my phone, but I can't see annotations on my phone because of software issue.

**Alice:** That limits communication/participation. Okay.

**Kate:** To access the doc and see content, I need my computer. Sometimes my computer is already put away, I'm too lazy to get it. Might be laziness.

**Alice:** During this annotated reading process, is there anything memorable?

**Kate:** This is not directly related to English. I think group annotated reading has strengthened our group communication and cooperation skills. Thanks to this and other group assignments, we have honed our teamwork and mutual understanding. Next time, we will automatically divide the work among ourselves.

**Alice:** So it helped with team building. Was it initially painful? Did it get easier later or stay painful?

**Kate:** It was quite painful throughout. The later Evaluative Annotation text was challenging. My vocabulary was limited, and my thinking was restricted. I always found it difficult. But compared to the first time, at least I knew the process and what to do.

**Alice:** When your group did annotated reading, did you discuss offline face-to-face or online via WeChat? Also, how did you choose the presenter for your two presentations (besides my random draw)? And do you listen carefully when other groups present? Do you take notes?

**Kate:** For the first question, last semester when we did annotated reading, we discussed offline face-to-face (since we're all roommates, face-to-face is more convenient). For the second question, besides the teacher's random draw, the first time we also drew lots. For the third question, I might pay less attention than some classmates, but I do focus on the presenter's content, locating the same spot in the text. If they say something interesting, like rewriting a sentence as an example, I'll note it down quickly.

**Alice:** Okay. I got new insights from you, different from others. Thank you. Could you add my WeChat? If I have follow-ups, I'll message you.

**Kate:** Okay, can do.

**Alice:** (Shares number) Okay, you can leave the meeting. I'll add you. Thanks!

**Kate:** Okay. Thanks for your hard work, teacher. Bye!
