# Supplementary material for: Developing students’ reading competence in an EFL context through annotated reading: A blended instructional design
Source: PLoS One. 2026 Oct 14;21(10):e0357895. doi: 10.1371/journal.pone.0357895 (PMC13667772; doi:10.1371/journal.pone.0357895)
Supplement: Supplementary file 3 [file pone.0357895.s003.docx]

**S1 Appendix**

Major questions for semi-structured interviews

1.Before this semester, had you ever been exposed to annotated reading?

2. What have you learned through annotated reading across this semester?

3.How did you conduct individual annotated reading and group discussion about annotated reading?

4.Have your reading methods/strategies changed across the semester?

5.Has annotated reading been helpful for your English reading studies?

6.Has there been any changes in your thinking/cognition?

7.What difficulties or challenges have you faced?

8.During the annotated reading process, was there anything that left a deep impression on you?
