# Supplementary material for: Developing students’ reading competence in an EFL context through annotated reading: A blended instructional design
Source: PLoS One. 2026 Oct 14;21(10):e0357895. doi: 10.1371/journal.pone.0357895 (PMC13667772; doi:10.1371/journal.pone.0357895)
Supplement: Supplementary file 4 [file pone.0357895.s006.docx]

Dear Editor, reviewers and referees,

Thank you very much for your careful review of our manuscript titled “Developing students’ reading competence in an EFL context through annotated reading: A blended instructional design” (PONE-D-25-59094). We appreciate your insightful comments and suggestions, which have significantly improved the quality of our work. Below is our detailed response to each comment, with corresponding revisions marked in the revised manuscript.

**Reviewer #1**: The manuscript is very well written. The authors have argued for the study convincingly. However, I just do not understand why they call it a quasi experimental study. Did I miss something? Did the study have a control group? If it did not have a control group, they should frame the study as a quasi experimental study. It is difficult to attribute the identified differences to the pedagogical approach used in the pedagogical intervention. Unless the authors could reframe the study, I am not sure if it is publishable in this journal.

Response: Thank you for your question. You raise a critical point regarding the classification of our study design. Upon re-examining the manuscript, we realize that the role of the control class was not described with sufficient clarity. Thank you for pointing out that this important detail needed to be highlighted. We have now clarified it in the revised manuscript.

To address your concern directly: Yes, our study does include a control group/class (referred to as CC).

To clarify how this constitutes a quasi-experimental design, please allow us to restate the design more explicitly:

Control Class (CC) and Experimental Class (EC) shared the same English teacher, the same course, as well as the same circumstances. The teacher taught the two classes using the same methods and materials, except for four target weeks (Weeks 3, 6, 10, and 13). On those weeks, the CC received traditional lecture-based instruction for the target texts, while the EC engaged in class reports focusing on annotations. We compared the pre-test and post-test scores between the EC and CC. The two classes were comparable at baseline (each with 54 students as stated in Lines 172-173, with no statistically significant difference in pre-test scores as shown in Table 5), the only systematic difference between them was the specific pedagogical approach applied to the target texts during those weeks. This controlled context further strengthens our ability to attribute any post-test differences to the intervention itself.

This design (non-random assignment of intact classes, with a non-equivalent control group) is the textbook definition of a quasi-experimental design.

In the revised manuscript, we have revised the “3.3 Research design” section (Lines 242-246) to more clearly describe the role of the CC, making the relationship between the groups clear. The revised part goes as follows, “As for the CC, they were identical to the EC in all respects (same teacher, same materials, same course structure) except that during the four target weeks, the CC received traditional lecture-based instruction on the target text while the EC engaged in class reports and feedback focusing on annotations. The pre-test and post-test for the EC were conducted on the same days and used the same papers as those for the CC.”

Thank you again for your thoughtful review, which has helped us significantly improve the clarity of our manuscript.

**Reviewer #2**: The manuscript examines a significant issue in English as a Foreign Language (EFL) pedagogy and introduces a blended four-stage annotated reading framework. While the study demonstrates potential for publication, several conceptual, methodological, and reporting concerns require resolution prior to acceptance.

The manuscript frequently asserts effectiveness, despite no significant difference between the groups.

• The results indicate that the post-test difference was not statistically significant (p = 0.32).

• Nevertheless, the discussion section continues to claim that the framework “has great potential” and “validates effectiveness.”

The quasi-experimental design lacks strong control:

• Only two classes are taught by the same teacher

• No random assignment

• Unequal attrition (42 CC vs. 51 EC)

• Limited intervention exposure (only four annotated texts)

The authors acknowledge that only 4 weeks of intervention occurred within a 16-week semester, which likely explains the non-significant results.

This limitation severely weakens causal inference.

Please consider the following manuscript to strengthen the theoretical framework of your work.

Öksüz, H. İ., Yaşartürk, İ., Yıldız, M., Yazıcı Arıcı, E., Sotiropoulos, D., & Kalogiannakis, M. (2026). Sustained Attention as a Cognitive Bridge Between Memory and Reading Comprehension in Primary School Students. Reading Psychology, 47(2), 276–303. <https://doi.org/10.1080/02702711.2025.2566025>

Response: Thank you for your insightful comment. We will divide your concerns into three parts and explain them one by one.

1. “The results indicate that the post-test difference was not statistically significant (*p* = 0.32). Nevertheless, the discussion section continues to claim that the framework ‘has great potential’ and ‘validates effectiveness’.”

Response :

Thank you for this valuable observation. We agree that the original wording was overly assertive given the non-significant quantitative results, and we have revised the manuscript accordingly.

We would like to clarify, however, that the claim of “potential” was based on two considerations. Firstly, although the post-test difference was not statistically significant, the experimental group showed a numerically larger increase in average post-test scores compared to the control group (see Fig 1). This descriptive trend suggests that the intervention may have promise for developing students’ reading competence. Secondly, as noted in the manuscript (Lines 108–114), reading competence is a multifaceted construct that includes cognitive abilities, metacognitive strategies, critical evaluation, and motivation and engagement. While the multiple-choice reading tests primarily captured cognitive abilities and critical evaluation, they could not adequately assess meta-cognitive growth, motivation or engagement. To address these unmeasured dimensions, we adopted semi-structured interviews. As shown in Table 9, the qualitative data indicated that interviewees benefited from the intervention across four themes: “Enhanced Cognition and Skills,” “Increased Motivation and Shifted Attitudes,” “Development of Metacognition and Self-Directed Learning,” and “Enhanced Application and Transfer Awareness”. Therefore, we now argue that it shows potential for further development and testing, particularly for fostering a more well-rounded reading competence..

That said, we also recognize that the original phrasing overstated the quantitative findings. We have therefore made the following revisions throughout the manuscript:

•Removed the word “great” from “great potential” (now simply “potential”).

•Deleted expressions such as “validates effectiveness” that directly imply statistical confirmation.

•Revised the conclusion (Lines 508–513) to read as follows:

“Although the quantitative results did not reach statistical significance, the qualitative data suggested students’ development across multiple sub-dimensions of reading competence. Furthermore, the findings reveal a meaningful pattern that modes of learning (e.g., paper-based annotated reading vs social reading; online vs face-to-face discussion) are not decisive factors in shaping learning outcomes. Instead, students’ engagement appears to play a pivotal role.”

We believe these revisions address the reviewer’s concern and present the findings with appropriate caution. Thank you again for this helpful critique.

2. The quasi-experimental design lacks strong control:

• Only two classes are taught by the same teacher

• No random assignment

• Unequal attrition (42 CC vs. 51 EC)

• Limited intervention exposure (only four annotated texts)

The authors acknowledge that only 4 weeks of intervention occurred within a 16-week semester, which likely explains the non-significant results.

Response: Thank you for the careful and constructive comments. We fully acknowledge the limitations of the quasi-experimental design and agree that they affect the strength of causal inference. However, we would like to clarify the contextual constraints and provide additional information to contextualize the design choices.

Firstly, due to institutional policies at the participating university, individual instructors are not permitted to alter class compositions, curricula, or teaching arrangements. The two classes were pre-existing intact groups. While this limits internal validity, we would like to note that the two classes had the same number of students at the beginning of the semester (both 54 students), and the pre-test scores showed no statistically significant difference between the two groups, indicating comparability at baseline despite the absence of random assignment.

Secondly, as for unequal attrition (42 CC vs. 51 EC), the difference in post-test sample size was primarily due to uncontrollable factors, including students transferring to other majors (a common occurrence at the beginning of the semester in this institution) and absence on the day of the post-test. These were not related to the intervention itself. The two groups started with equal enrollment (54 each), and the attrition was largely exogenous. We have now acknowledged this as a limitation in the revised manuscript (Lines 531-533 ): Besides, even though the unequal attrition (42 in the CC vs 51 in the EC) was largely exogenous, the imbalance may still introduce potential bias. To mitigate this concern, future studies should carefully estimate expected attrition rates a priori and recruit a larger initial sample accordingly.

At last, we agree that the intervention length is limited. However, this was the maximum duration permissible within the existing 16-week semester structure without disrupting the required curriculum. We have explicitly stated in the manuscript that the non-significant results are likely attributable to the brief intervention period (Line 441-446).

Thank you again for the helpful critique, which has significantly improved the clarity and honesty of our manuscript.

3. Please consider the following manuscript to strengthen the theoretical framework of your work.

Öksüz, H. İ., Yaşartürk, İ., Yıldız, M., Yazıcı Arıcı, E., Sotiropoulos, D., & Kalogiannakis, M. (2026). Sustained Attention as a Cognitive Bridge Between Memory and Reading Comprehension in Primary School Students. Reading Psychology, 47(2), 276–303. <https://doi.org/10.1080/02702711.2025.2566025>

Response 3: Thank you for suggesting the insightful work of Öksüz et al. (2026). We agree that their conceptualization of Sustained Attention (SA) as a cognitive bridge between memory and reading comprehension provides a strong theoretical grounding for our instructional design.

Following your suggestion, we have incorporated this reference into our theoretical framework. Specifically, on Lines 76–82, we now explicitly position SA as the core cognitive underpinning of annotated reading, and we cite Öksüz et al. (2026) to support the claim. The added text reads as follows: Sustained Attention (SA) can be conceptualized as the core cognitive underpinning for annotated reading. Öksüz et al. [21] demonstrate that SA serves as a “cognitive bridge” during reading—it not only directly predicts comprehension levels but also acts as a full mediator, transforming the efficacy of verbal working memory into accessible reading comprehension ability. Thus, the practice of annotated reading—which requires readers to continuously select, mark, and reflect on text segments—can be seen as a natural behavioral expression of SA, training the very process of consistently and strategically allocating attention to key information.

We believe this addition sharpens the theoretical clarity of our manuscript and better justifies why our four-stage annotated reading instruction—which trains sustained and strategic allocation of attention—is pedagogically sound. We are grateful for your constructive guidance.

**Reviewer #3**: This manuscript addresses a timely and pedagogically relevant topic by examining whether a blended annotated reading design can support the development of EFL students’ reading competence. The attempt to move beyond purely online annotation and to incorporate students’ perceptions and actualization is valuable. The study also has the potential to contribute to ongoing discussions about technology use, learner engagement, and the broader teaching-learning process. However, the current version still requires several revisions before publication can be considered.

## **Comment 1:**

Your manuscript is situated within the Chinese EFL context; however, the broader significance and scale of this setting are not yet immediately clear to international readers. At present, the manuscript provides some useful local contextual information. For example, the participants are described as “**108 second-year non-English major undergraduates**” in “**a south China university**” taking **College English III**, and their English proficiency is briefly characterized through **CET-4**. While these details are helpful, they mainly describe the immediate classroom setting **rather than the wider Chinese EFL landscape**. I therefore suggest strengthening the Method or Introduction section by adding a concise overview of the scale and significance of English language learning in China. This will help readers better appreciate both the broader relevance and the transferability of your study.

For example, you can write something like: “China had already surpassed 390 million English learners/users by 2000 (Wei & Su, 2012), and subsequent work (e.g. Wei & Gao, 2022) suggests the continued growth of English learning in China.” Such contextualization would make the study’s contribution more accessible and meaningful to an international readership.

## **References:**

Wei, R. & Gao, X. (2022). New research trends in TESOL: An introduction. *Chinese*

*Journal of Applied Linguistics, 45*(2), 220-223.

Wei, R. & Su, J. (2012). The statistics of English in China: An analysis of the best available data from government sources. *English Today, 28* (3), 10-14.

Response: Thank you for your thoughtful and constructive suggestion. We agree that providing a broader contextual overview of English language learning in China will help international readers better appreciate the scale, relevance, and transferability of our study.

In response to your comment, we have added the following concise contextualization in the Method section (immediately after describing the participants, Lines 179-188) , citing the references you provided:

To contextualize the scale and significance of English language learning in China, it is noteworthy that the country had already surpassed 390 million English learners and users by 2000 [42], and subsequent research (e.g., [43]) indicates continued growth in the English learning population. However, despite over ten years of English instruction, the participants in this study exhibited lower to intermediate proficiency, with nearly half having only just passed the College English Test Band 4 (CET4, approximately equivalent to A2 on the CEFR). This proficiency profile is common among a substantial segment of English learners in China, highlighting the practical relevance of this study for understanding instructional interventions in large-scale, compulsory English education contexts. Such contextualization enhances the transferability of the findings to similar educational settings, both within and beyond China.

## **Comment 2:**

You are requested to consistently use the term ‘statistically significant’ instead of ‘significant’. The omission of the term ‘statistically’ can easily result in the erroneous impression that the results are important. Hence many scholars (e.g. Carver, 1993) have underlined that the term ‘statistically’ must always precede the word ‘significant’.

## **References:**

Carver, R. P. (1993). The case against statistical significance testing, revisited. *The Journal of Experimental Education, 61*(4), 287–292.

Response: Thank you for this important methodological reminder. We fully agree that the term “statistically” should precede “significant” to avoid any potential confusion. Following your suggestion, we have carefully reviewed the quantitative results and related discussion of our manuscript and added “statistically” before corresponding occurrence of “significant” or “nonsignificant.”

We appreciate your attention to this terminological precision, which has strengthened the clarity and rigor of our manuscript.

## **Comment 3:**

Echoing Comment 2 about the over-reliance on the statistical significance level (viz. *p*), this comment addresses the same issue in your manuscript regarding the use of **effect sizes**. In the quantitative section, the manuscript reports Mann-Whitney U test results mainly in terms of *p* values (e.g., ***p* = .83 and *p* = .32** in Line 320 and Line 327), but **no corresponding effect sizes are provided.** This is not sufficient. Best reporting practice in applied linguistics and beyond requires researchers to report effect sizes for **both statistically significant and non-significant results** (Larson-Hall & Plonsky, 2015; Wei et al., 2024). In other words, *p* values alone do not tell readers how large, small, or practically meaningful the observed differences are. Effect sizes are therefore indispensable, as they provide crucial information about the magnitude of the observed effect and facilitate more meaningful interpretation and comparison across studies.

I strongly recommend that you adequately report and interpret effect sizes throughout

the quantitative section. For the Mann–Whitney *U* tests, an *r*-family effect size can be reported; for any *t* tests used, Cohen’s *d* should also be reported and interpreted based **on an explicit benchmark system**. At present, the manuscript’s statistical interpretation remains overly *p*-value-driven.

If you need good examples of the reporting and interpretation of effect sizes, please consult Wang & Wei (2024) and Shen and Wei (2025).

References:

Larson-Hall, J., & Plonsky, L. (2015). Reporting and interpreting quantitative research findings: What gets reported and recommendations for the field. *Language Learning, 65*(S1), 127–159.

Shen, J., & Wei, R. (2025). Is multilingualism linked to attitudes toward gender equality? Evidence from a big data survey. *Journal of Multilingual and Multicultural Development*. Advance online publication. https://doi.org/10.1080/01434632.2025.2552327

Wang, J., & Wei, R. (2024). Is bilingualism linked to well-being? Evidence from a big- data survey. *Bilingualism: Language and Cognition, 27*, 546–556. https://doi.org/10.1017/S1366728923000603

Wei, R., Reynolds, B. L., Kong, M., & Liu, Z. (2024). Is bilingualism linked to national identity? Evidence from a big data survey. *Journal of Multilingual and Multicultural Development, 45*(8), 3078–3092. <https://doi.org/10.1080/01434632.2022.2085282>

Response: Thank you for this constructive and important comment. We agree that over-reliance on statistical significance (*p* values) is insufficient and that effect sizes should be reported and interpreted for the quantitative results.

In response to this comment, we have now calculated and reported the *r*-family effect sizes for the Mann-Whitney U tests. These have been added to Table 5 and Table 6. Specifically, we report the effect sizes for the comparisons between the experimental group (EC) and the control group (CC). These additions have also been described in the revised manuscript (see Lines 297-299).

As shown in the revised tables, the effect size increased from *r* = 0.02 at pre-test to *r* = 0.10 at post-test. Although the post-test difference did not reach statistical significance (*p* = 0.32), the small but non-negligible effect size (*r* = 0.10) suggests that the instructional intervention may have a limited but practical potential in improving students’ reading competence. This interpretation follows Cohen’s (1988) conventions, where *r* = 0.10 is considered a small effect.

We believe that the revised manuscript now provides a more balanced and informative statistical interpretation, moving beyond a purely p-value-driven approach. Once again, we appreciate your valuable suggestion and the articles provided for our reference.

References:

Cohen, J. (1988). *Statistical power analysis for the behavioral sciences* (2nd ed.). Psychology Press/Taylor & Francis Group.

## **Comment 4:**

The analysis for Fig. 5 (Line 339-344) appears to be a simple **individual gain-score analysis** based on each student’s post-test minus pre-test score in the EC. In other words, the classification of “**39 students improved, 7 declined, and 5 showed no change**” (Line 340) seems to be purely descriptive. However, although the Methods section states that “paired samples t-test checked pre-test-post-test differences within the EC and CC” (Line 278), the Results section does not appear to report those inferential results. Please clarify whether such within-group tests were actually conducted and, if so, report them fully. If the distributional assumptions were not met, a nonparametric within-group alternative may be more appropriate.

This issue should also be corrected in the revised manuscript. Importantly, the pre-test- post-test differences within the EC and CC are not a trivial detail, as they are necessary for understanding whether each group changed over time and for interpreting the intervention effect more rigorously in a quasi-experimental pre-test/post-test design.

Response: Thank you for your careful observation. We apologise for the lack of clarity in the original manuscript regarding the within-group inferential analyses.

To address your comment, we have now fully reported the paired-samples *t*-test for the CC and the Wilcoxon signed-rank test for the EC in the revised manuscript.

For the CC, a paired-samples *t*-test was conducted, which showed a statistically significant improvement from pre-test to post-test (*t* = -2.37, *p* = .023, Cohen’s *d* = -0.37, 95% CI [-0.68, -0.05]), indicating a small-to-medium effect.

For the EC, because of the conservative approach to normality, a Wilcoxon signed-rank test was performed, revealing a statistically significant improvement (*Z* = -3.844, *p* < .001, *r* = .54, 95% CI [0.30, 0.70]), indicating a large effect.

These results have been added to the Results section (see Table 7 and Table 8, and corresponding text in Lines 308–326). The descriptive gain-score analysis of the EC (39 improved, 7 declined, 5 no change) in Fig 2 is presented as a supplementary descriptive illustration following the inferential statistical results, rather than as a substitute for them.

We believe the revised manuscript now clearly demonstrates that both groups showed statistically significant improvements over time, with a substantially larger effect size in the EC due to the pedagogical intervention. We greatly appreciate your constructive feedback, which has helped us strengthen the rigour and clarity of our statistical reporting.

**As for the journal requirements, we have done as required.**

1. Please ensure that your manuscript meets PLOS ONE’s style requirements, including those for file naming. The PLOS ONE style templates can be found at

https://journals.plos.org/plosone/s/file?id=wjVg/PLOSOne_formatting_sample_main_body.pdf  and https://journals.plos.org/plosone/s/file?id=ba62/PLOSOne_formatting_sample_title_authors_affiliations.pdf

Response: We have carefully reviewed the manuscript to ensure it meets PLOS ONE’s style requirements. Corresponding revisions have been made.

2. Thank you for stating the following financial disclosure:

“This research has been founded by Department of Education of Guangdong Province, China in 2024.”

Please state what role the funders took in the study.  If the funders had no role, please state: "The funders had no role in study design, data collection and analysis, decision to publish, or preparation of the manuscript." If this statement is not correct you must amend it as needed.

Please include this amended Role of Funder statement in your cover letter; we will change the online submission form on your behalf.

Response: Thank you for your reminder. The following statement has been included at the end of our cover letter: “The funders had no role in study design, data collection and analysis, decision to publish, or preparation of the manuscript.”

3.We note that Figures 1 and 3 in your submission contain copyrighted images. All PLOS content is published under the Creative Commons Attribution License (CC BY 4.0), which means that the manuscript, images, and Supporting Information files will be freely available online, and any third party is permitted to access, download, copy, distribute, and use these materials in any way, even commercially, with proper attribution. For more information, see our copyright guidelines: [http://journals.plos.org/plosone/s/licenses-and-copyright](http://journals.plos.org/plosone/s/licenses-and-copyright).
 
We require you to either (1) present written permission from the copyright holder to publish these figures specifically under the CC BY 4.0 license, or (2) remove the figures from your submission:
 
1) You may seek permission from the original copyright holder of Figures 1 and 3 to publish the content specifically under the CC BY 4.0 license. 
 
We recommend that you contact the original copyright holder with the Content Permission Form ([http://journals.plos.org/plosone/s/file?id=7c09/content-permission-form.pdf](http://journals.plos.org/plosone/s/file?id=7c09/content-permission-form.pdf)) and the following text:
“I request permission for the open-access journal PLOS ONE to publish XXX under the Creative Commons Attribution License (CCAL) CC BY 4.0 ([http://creativecommons.org/licenses/by/4.0/](http://creativecommons.org/licenses/by/4.0/)). Please be aware that this license allows unrestricted use and distribution, even commercially, by third parties. Please reply and provide explicit written permission to publish XXX under a CC BY license and complete the attached form.”
 
Please upload the completed Content Permission Form or other proof of granted permissions as an ""Other"" file with your submission. 
 
In the figure caption of the copyrighted figure, please include the following text: “Reprinted from [ref] under a CC BY license, with permission from [name of publisher], original copyright [original copyright year].”
 
2) If you are unable to obtain permission from the original copyright holder to publish these figures under the CC BY 4.0 license or if the copyright holder’s requirements are incompatible with the CC BY 4.0 license, please either i) remove the figure or ii) supply a replacement figure that complies with the CC BY 4.0 license. Please check copyright information on all replacement figures and update the figure caption with source information. If applicable, please specify in the figure caption text when a figure is similar but not identical to the original image and is therefore for illustrative purposes only.

Response: Thank you for your guidance. After careful consideration, we have decided to remove Figures 1, 2, and 3 from our manuscript. While your comment specifically noted Figures 1 and 3, we have also removed Figure 2 because it is a screenshot showing the function of posting pictures or videos on Tencent Document, serving a similar purpose as Figures 1 and 3 in illustrating the features of this software. Furthermore, given that Figures 1 and 3 have been removed, retaining Figure 2 alone would disrupt the logical flow and coherence of the presentation. Therefore, we have removed all three figures. All in-text references to these figures have been deleted, and the remaining figures have been renumbered accordingly. We appreciate your understanding.

4. Please include captions for your Supporting Information files at the end of your manuscript, and update any in-text citations to match accordingly. Please see our Supporting Information guidelines for more information: [http://journals.plos.org/plosone/s/supporting-information](http://journals.plos.org/plosone/s/supporting-information).

Response: Thank you for your reminder. In accordance with the journal’s Supporting Information guidelines, we added a “Supporting Information Captions” section at the end of the revised manuscript. The captions are listed as follows:

S1 Table. Complete quantitative dataset (Excel file).

S1 Text. Anonymized interview transcripts (English version).

S1 Appendix. Major questions for semi-structured interviews.

We have also carefully reviewed and updated all in-text citations to match these labels accordingly. We confirm that the manuscript now complies with the requirements for supporting information.

5. Thank you for providing your underlying data as Supporting Information. We note that the data set contains text or data that is not in English. Please note that PLOS is an English-language publisher, so we require data sets to be provided in English as well. Please upload an English-language version of your data set. This will also allow us to determine if your data follows PLOS standards per our Data Availability policy here: https://journals.plos.org/plosone/s/data-availability

Response: Thank you for your reminder. We have addressed the language issue for our Supporting Information. The interview transcripts, which were originally not in English, have now been translated into English and are designated as S1 Text. Both the translated interview transcripts and the quantitative dataset will be re-uploaded to the submission system.

6. We note that this data set consists of interview transcripts. Can you please confirm that all participants gave consent for interview transcript to be published?

If they DID provide consent for these transcripts to be published, please also confirm that the transcripts do not contain any potentially identifying information (or let us know if the participants consented to having their personal details published and made publicly available). We consider the following details to be identifying information:
- Names, nicknames, and initials
- Age more specific than round numbers
- GPS coordinates, physical addresses, IP addresses, email addresses
- Information in small sample sizes (e.g. 40 students from X class in X year at X university)
- Specific dates (e.g. visit dates, interview dates)
- ID numbers

Or, if the participants DID NOT provide consent for these transcripts to be published:
- Provide a de-identified version of the data or excerpts of interview responses
- Provide information regarding how these transcripts can be accessed by researchers who meet the criteria for access to confidential data, including:
a) the grounds for restriction
b) the name of the ethics committee, Institutional Review Board, or third-party organization that is imposing sharing restrictions on the data
c) a non-author, institutional point of contact that is able to field data access queries, in the interest of maintaining long-term data accessibility.
d) Any relevant data set names, URLs, DOIs, etc. that an independent researcher would need in order to request your minimal data set.

For further information on sharing data that contains sensitive participant information, please see: [https://journals.plos.org/plosone/s/data-availability#loc-human-research-participant-data-and-other-sensitive-data](https://journals.plos.org/plosone/s/data-availability#loc-human-research-participant-data-and-other-sensitive-data)

If there are ethical, legal, or third-party restrictions upon your dataset, you must provide all of the following details ([https://journals.plos.org/plosone/s/data-availability#loc-acceptable-data-access-restrictions](https://journals.plos.org/plosone/s/data-availability#loc-acceptable-data-access-restrictions)):
1. A complete description of the dataset
2. The nature of the restrictions upon the data (ethical, legal, or owned by a third party) and the reasoning behind them
3. The full name of the body imposing the restrictions upon your dataset (ethics committee, institution, data access committee, etc)
4. If the data are owned by a third party, confirmation of whether the authors received any special privileges in accessing the data that other researchers would not have
5. Direct, non-author contact information (preferably email) for the body imposing the restrictions upon the data, to which data access requests can be sent

Response: Thank you for your request. Regarding the interview transcripts provided as Supporting Information, we confirm the following:

All participants gave verbal consent for their interview transcripts to be published.

The transcripts have been carefully reviewed and do not contain any potentially identifying information (such as names, nicknames, initials, specific ages, specific dates, addresses, contact details, ID numbers, or any information that could lead to identification through small sample sizes).

7.If the reviewer comments include a recommendation to cite specific previously published works, please review and evaluate these publications to determine whether they are relevant and should be cited. There is no requirement to cite these works unless the editor has indicated otherwise. 
Response: We greatly appreciate the reviewers’ valuable suggestions regarding the citation of previously published works. We have carefully reviewed and evaluated all the recommended publications. Some of these works are indeed relevant to our study and have been cited accordingly in the revised manuscript. For the remaining recommended publications, we have taken them into consideration but, based on our assessment, they are not directly applicable to the current work. Nonetheless, we sincerely thank the reviewers for bringing these publications to our attention.
