# Supplementary material for: Developing students’ reading competence in an EFL context through annotated reading: A blended instructional design
Source: PLoS One. 2026 Oct 14;21(10):e0357895. doi: 10.1371/journal.pone.0357895 (PMC13667772; doi:10.1371/journal.pone.0357895)
Supplement: Supplementary file 5 [file pone.0357895.s007.docx]

Dear Reviewer #2,

We would like to express our sincere gratitude for your thoughtful and constructive feedback on our manuscript. We are delighted that you consider our research topic relevant and the proposed pedagogical framework valuable.

With regard to your comment on ethical considerations, we fully acknowledge the necessity of an explicit ethical declaration. Accordingly, we have supplemented a detailed ethical statement in the revised manuscript (Lines 209–218).

Following your recommendation, the article you suggested has also been included in our reference list.

We believe these revisions effectively strengthen our manuscript by elaborating on the critical ethical dimension of this study. We hope these adjustments satisfy your requirements.

Thank you again for your time and insightful suggestions.

Sincerely,

Haoming Zhang
